# Endothelial progerin expression causes cardiovascular pathology through an impaired mechanoresponse

**DOI:** 10.1172/JCI121297

**Published:** 2018-12-18

**Authors:** Selma Osmanagic-Myers, Attila Kiss, Christina Manakanatas, Ouafa Hamza, Franziska Sedlmayer, Petra L. Szabo, Irmgard Fischer, Petra Fichtinger, Bruno K. Podesser, Maria Eriksson, Roland Foisner

**Affiliations:** 1 Max F. Perutz Laboratories (MFPL), Department of Medical Biochemistry, Medical University of Vienna and University of Vienna, Vienna Biocenter (VBC), Vienna, Austria.; 2 Department of Biotechnology, University of Natural Resources and Life Sciences (BOKU), Vienna, Austria.; 3 Ludwig Boltzmann Cluster for Cardiovascular Research at the Center for Biomedical Research, Medical University of Vienna, Vienna, Austria.; 4 Department of Biosciences and Nutrition, Karolinska Institutet, NEO, Huddinge, Sweden.

**Keywords:** Aging, Cell Biology, Fibrosis, Mouse models, endothelial cells

## Abstract

Hutchinson-Gilford progeria syndrome (HGPS) is a premature aging disorder characterized by accelerated cardiovascular disease with extensive fibrosis. It is caused by a mutation in *LMNA* leading to expression of truncated prelamin A (progerin) in the nucleus. To investigate the contribution of the endothelium to cardiovascular HGPS pathology, we generated an endothelium-specific HGPS mouse model with selective endothelial progerin expression. Transgenic mice develop interstitial myocardial and perivascular fibrosis and left ventricular hypertrophy associated with diastolic dysfunction and premature death. Endothelial cells show impaired shear stress response and reduced levels of endothelial nitric oxide synthase (eNOS) and NO. On the molecular level, progerin impairs nucleocytoskeletal coupling in endothelial cells through changes in mechanoresponsive components at the nuclear envelope, increased F-actin/G-actin ratios, and deregulation of mechanoresponsive myocardin-related transcription factor-A (MRTFA). MRTFA binds to the *Nos3* promoter and reduces eNOS expression, thereby mediating a profibrotic paracrine response in fibroblasts. MRTFA inhibition rescues eNOS levels and ameliorates the profibrotic effect of endothelial cells in vitro. Although this murine model lacks the key anatomical feature of vascular smooth muscle cell loss seen in HGPS patients, our data show that progerin-induced impairment of mechanosignaling in endothelial cells contributes to excessive fibrosis and cardiovascular disease in HGPS patients.

## Introduction

Hutchinson-Gilford progeria syndrome (HGPS) is a rare, progressive, premature aging disease characterized by growth retardation, alopecia, loss of subcutaneous fat, aged-looking skin, bone abnormalities, and osteoporosis (1–3). A hallmark of HGPS is severe atherosclerosis in childhood characterized by left ventricular (LV) diastolic dysfunction associated with LV hypertrophy (4, 5). Cardiovascular features in HGPS include a spectrum of early- to late-stage collagen-rich plaques with a primary vascular pathology of severe vascular smooth muscle cell (VSMC) loss in large arteries (4, 6, 7) and massive fibrosis in the adventitial layer (4), hyaluronan accumulation, and calcification of blood vessels (8). These changes increase aortic stiffness and cardiac afterload, presumably causing LV hypertrophy and myocardial infarction in patients at a mean age of 14.6 years (9).

HGPS is caused by mutations in *LMNA*, encoding lamin A and C. Lamins are major components of the nuclear lamina, a complex protein meshwork underlying the inner nuclear membrane that provides shape and mechanical stability to the nucleus and interacts with heterochromatin (10). Lamin A is expressed as prelamin A and posttranslationally processed in a multistep pathway, including C-terminal farnesylation and carboxymethylation, followed by proteolytic cleavage of the modified C-terminal 15 amino acids, yielding nonfarnesylated and noncarboxymethylated mature lamin A (10). HGPS is predominantly caused by a dominant 1824C>T mutation in *LMNA* that activates a cryptic splice donor site leading to the formation of an incompletely processed and stably farnesylated prelamin A with a 50–amino acid internal deletion, termed progerin (11, 12).

HGPS-linked cardiovascular features resemble in many aspects those of geriatric cardiovascular disease (CVD) (4, 13–16). Despite the fact that endothelial senescence is a major initiating event in geriatric CVD and atherosclerosis (13, 17), research on CVD in HGPS has mainly focused on VSMCs based on the reports on VSMC depletion in HGPS patients and some murine models (4, 6, 8, 18, 19). The relevance of progerin expression in the endothelium and its contribution to cardiovascular pathologies in HGPS is still unknown. Aging-related changes in the endothelium involve downregulation of atheroprotective pathways such as reduced endothelial NO synthase (eNOS) levels, shear stress insensitivity (20), and activation of proinflammatory molecules ICAM1 and ICAM2 (17) increasing atherosclerosis susceptibility (21, 22). Other key features of aged, dysfunctional arteries include increased stiffening due to structural changes in the extracellular matrix (ECM) within intimal, medial, and adventitial layers (23), which in turn affect actomyosin-mediated cellular contractility and shear stress response (20, 23).

Only a few studies provide potential mechanistic links between progerin expression and CVD (24, 25). Gene expression profiling in HGPS patient fibroblasts revealed prominent misregulation of ECM genes (26, 27). In addition, progerin expression in cultured cells disturbed nucleocytoskeletal connections and affected the linker of nucleoskeleton and cytoskeleton (LINC) complex (28, 29), suggesting that defective mechanotransduction may play a role in HGPS. Various HGPS mouse models were generated, which show phenotypes that range from severe, leading to premature death (18, 30), to relatively mild (19), and several conditional HGPS mouse models have reported various tissue-specific effects of progerin expression (31–33). In regard to CVD, a recently reported mouse model with specific vascular smooth muscle expression of progerin presented VSMC loss and accelerated atherosclerosis (6), but hitherto endothelium-specific HGPS mice have not been reported. Based on the importance of age-related changes of the endothelium in CVD we generated a transgenic mouse model with progerin expression selectively in the endothelium (*Prog-Tg*). *Prog-Tg* mice develop LV hypertrophy and diastolic dysfunction, and myocardial interstitial and perivascular fibrosis, phenocopying many aspects of the HGPS cardiovascular pathology. Our data suggest that progerin expression in endothelial cells (ECs) causes severe cardiovascular pathology through deregulated antifibrotic pathways but does not cause VSMC loss. Mechanistically, progerin expression impaired nucleocytoskeletal coupling and shear stress response in ECs leading to impaired mechanoresponsive myocardin-related transcription factor A (MRTFA) signaling.

## Results

### Endothelium-specific expression of progerin leads to cardiovascular pathology.

**Figure 1:**
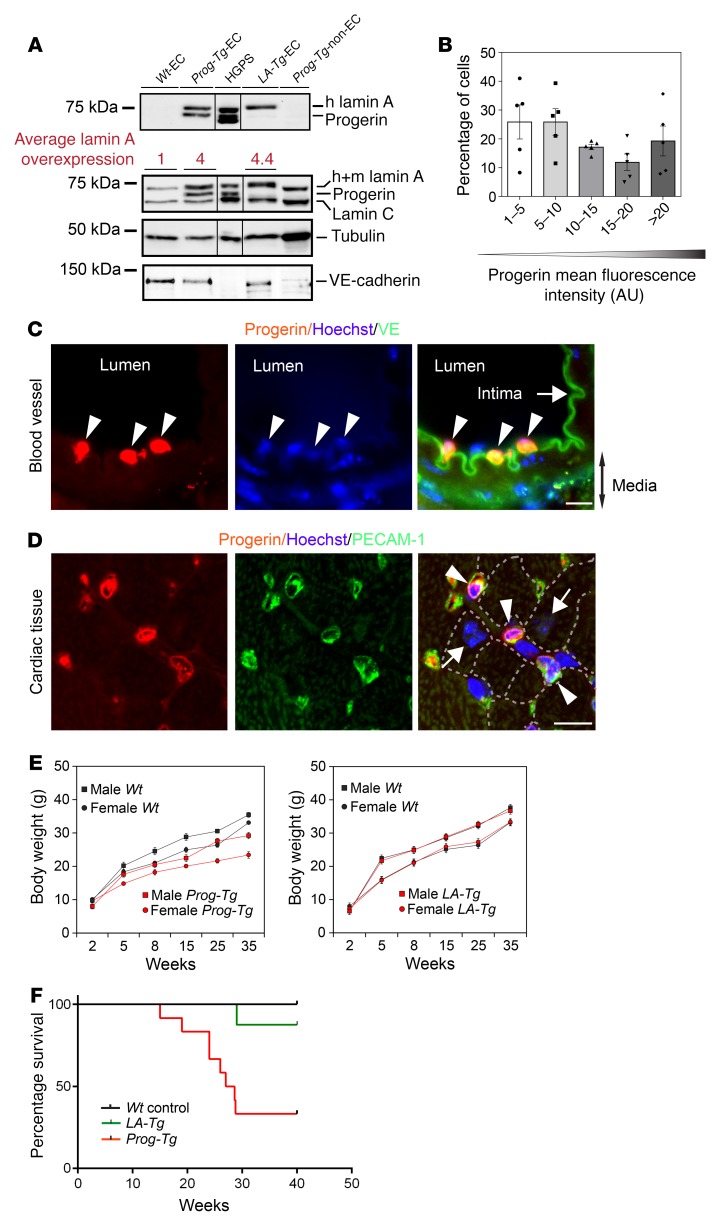
Characterization of *Prog-Tg* mice. (**A**) Immunoblots of indicated lysates using anti–human lamin A/C (detecting transgenes, upper panel), anti–lamin A/C (recognizing human and mouse lamin A/C, lower panel), and anti-tubulin and anti–VE-cadherin as loading controls. Numbers show ratio of total lamin A levels in transgenic over *Wt* animals. Endothelial cells (ECs) from *Wt* (*Wt*-EC), *Prog-Tg* (*Prog-Tg*-EC) and *LA-Tg* (*LA-Tg*-EC) animals; EC-depleted cell mixture from *Prog-Tg* animals (*Prog-Tg*-non-EC); and HGPS patient fibroblasts (HGPS) were analyzed. (**B**) Histogram of mean progerin fluorescence intensities in immunofluorescence images of *Prog-Tg* ECs (see Figure 5C) (*n* = 5 *Prog-Tg* mice, 462 cells in total). (**C** and **D**) Immunofluorescence images of coronary artery (**C**) and cardiac tissue (**D**) from *Prog-Tg* animals stained with antibodies against progerin, VE-cadherin (note green autofluorescence of elastic lamina), and PECAM1, and Hoechst (representative of *n* = 3 *Prog-Tg* animals). Progerin expression is confined to the intimal layer (**C**, arrowheads) and PECAM1-positive cardiac microvasculature (**D**, arrowheads). Dashed lines, cardiomyocyte boundaries; arrows, intima (**C**) and progerin-negative cardiomyocytes (**D**). Scale bars: 10 μm. (**E**) Body weight over time for male and female *Prog-Tg* and *LA-Tg* versus *Wt* littermates. Two-way repeated-measures ANOVA revealed a significant impact for the *Prog-Tg* genotype (females *F* = 72.6, *P* *<* 0.001, males *F* = 65.3, *P* < 0.001, *n* = 6 littermate pairs), but not for the *LA-Tg* genotype (females *F* = 0.517, *P* = 0.493; males *F* = 0.221, *P* = 0.651, *n* = 5 littermate pairs). Comparison of *Prog-Tg* versus *Wt* revealed at least *P* < 0.01 (Holm-Sidak method) for females and males at more than 5 and 8 weeks, respectively. (**F**) Kaplan-Meier survival plot showing significantly reduced life span of *Prog-Tg* mice (*n* = 12) versus *Wt* littermate controls (*n* = 20) and *LA-Tg* mice (*n* = 8). *P* < 0.0001, log-rank (Mantel-Cox) test; pairwise comparison with Bonferroni’s correction of threshold showed significant difference in survival of *Prog-Tg* compared with control, *Prog-Tg* with *LA-Tg*, but not *LA-Tg* with control mice. Data presented as mean ± SEM.

To determine the impact of progerin expression in the endothelium on cardiovascular pathology in HGPS, we established mice expressing progerin exclusively in ECs by crossing transgenic mice carrying tet operon–driven wild-type or HGPS mutant (1824C>T; G608G) lamin A minigenes (32), with transgenic mice expressing a tetracycline-responsive transcriptional activator under the control of the endothelium-specific *Cdh5* promoter (34). Bitransgenic mice expressing human progerin and wild-type lamin A in endothelial tissue are referred to as *Prog-Tg* and *LA-Tg* mice, respectively, and their corresponding single-transgenic *tetop-LA^Wt^* and *tetop-LA^G608G^* littermates as *Wt*. To verify endothelium-specific expression, we performed immunoblot analyses of EC and non-EC extracts (see Methods) using human lamin A–specific antibodies that do not detect endogenous mouse lamin A. As previously shown (32), the HGPS minigene in *Prog-Tg* mice expressed both the ectopic wild-type human lamin A and progerin in ECs but not in non-ECs, while the wild-type lamin A minigene in *LA-Tg* mice expressed only wild-type lamin A (Figure 1A). *Wt* mice did not express human ectopic lamin A proteins in ECs, confirming a tight regulation from the tet operon. Immunoblots using an antibody detecting both endogenous and ectopic lamin A indicated a 4- and 4.4-fold increase in total lamin A levels in *Prog-Tg* and *LA-Tg* versus *Wt* animals, respectively. Immunofluorescence staining of a *Prog-Tg* lung–derived cell mixture using human lamin A antibodies confirmed exclusive expression of transgenes in endothelial, vascular endothelial cadherin–positive (VE-cadherin–positive) cells (Supplemental Figure 1A; supplemental material available online with this article; https://doi.org/10.1172/JCI121297DS1). Mean fluorescence intensity measurement of progerin signals revealed heterogeneous expression levels among individual cells, as reported in HGPS patients (35), with over 75% of nuclei showing clearly detectable progerin staining (Figure 1B). Furthermore, in cardiac coronary vessels, progerin staining was specifically detected in the intimal but not medial and adventitial layers of the artery (Figure 1C and Supplemental Figure 1B, arrowheads). In cardiac tissue, progerin is only expressed in the PECAM1-positive microvasculature (Figure 1D, arrowheads), but not in cardiomyocytes (arrows). Overall, these data confirmed a tightly controlled, endothelium-specific expression of the transgenes in bitransgenic mice.

**Figure 2:**
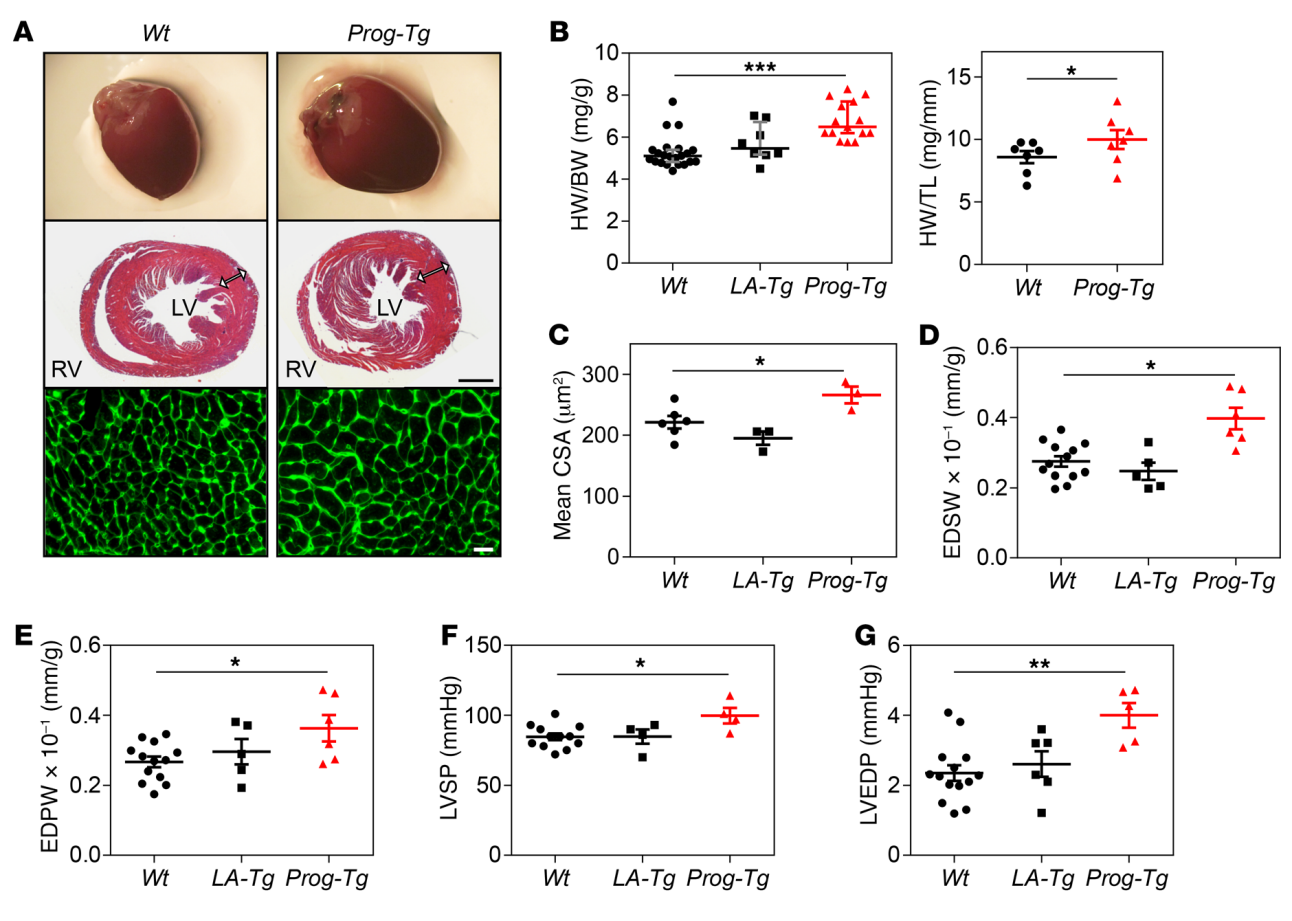
Cardiac hypertrophy and diastolic dysfunction in *Prog-Tg* mice. (**A**) Representative heart images (upper panel) and H&E-stained thin slices of cardiac ventricle cross sections of *Wt* and *Prog-Tg* mice at 6 months (*n* = 3 per genotype). Arrows indicate thickness of left ventricular free wall. Scale bar: 1,000 μm. High-power view of left ventricular wall area (lower panel) using cell membrane staining with wheat germ agglutinin–fluorescein conjugate for cardiomyocyte area analysis (*n* = 3 per genotype). Scale bar: 20 μm. (**B**) Diagrams depict heart weight to body weight (HW/BW) ratio in *Wt*, *LA-Tg*, and *Prog-Tg* mice (age >25 weeks, *n* = 22, 8, and 15 mice, respectively, Kruskal-Wallis 1-way ANOVA). Multiple comparisons (Dunn’s method) versus *Wt* control group reveal ***P < 0.001 for *Prog-Tg* and not significant for *LA-Tg* mice. Data presented as values (dots), median (middle line), and whiskers (25th and 75th percentile). Right panel shows HW to tibia length (TL) ratio in *Wt* and *Prog-Tg* mice (*n* = 7 littermates; age >27 weeks). *P < 0.05 by paired Student’s *t* test. Data presented as mean ± SEM. (**C**) Mean cardiomyocyte cross-sectional area (CSA), *n* = 6 for *Wt* and *n* = 3 mice for *LA-Tg* and *Prog-Tg* genotypes, >2,000 cells per genotype. (**D**) End-diastolic septal wall width to body weight ratio (EDSW). (**E**) End-diastolic posterior wall width to body weight ratio (EDPW). (**F**) Left ventricular systolic pressure (LVSP). (**G**) Left ventricular end-diastolic pressure (LVEDP). For **D**–**G**, age >27 weeks, *n* = 11 *Wt*, *n* = 5 *LA-Tg*, *n* = 6 *Prog-Tg*. For **C**–**G**, 1-way ANOVA, pairwise comparisons versus *Wt* control group, *P < 0.05 or **P < 0.01 for *Prog-Tg* and not significant for *LA-Tg* mice (Holm-Sidak method). Data presented as mean ± SEM.

Next we analyzed the phenotype of bitransgenic mice. We observed reduced growth of male and female *Prog-Tg* but not *LA-Tg* mice (Figure 1E) compared with *Wt* littermates, and in approximately 40% of *Prog-Tg* but not *LA-Tg* mice sudden death occurred at the age approaching 25 weeks (Figure 1F). Since myocardial infarction is the most common cause of death in HGPS patients (4), we examined the hearts of bitransgenic mice. Increased heart weight to body weight ratios in *Prog-Tg* versus *Wt* littermates and *LA-Tg* animals as well as increased heart weight to tibia length ratios in *Prog-Tg* versus *Wt* mice (Figure 2, A and B) indicated cardiac hypertrophy in *Prog-Tg* mice. Histological examination of heart sections showed thickened left ventricles in *Prog-Tg* versus *Wt* littermates (Figure 2A) and an increased mean cross-sectional area of cardiomyocytes (Figure 2, A and C). Unlike *NppA*, expression of hypertrophy marker *NppB* (36, 37) showed a tendential increase in heart and a significant increase in isolated primary *Prog-Tg* ECs and lung (Supplemental Figure 2A). We next performed transthoracic cardiac echocardiography and subsequent LV pressure characterization to examine cardiac functional parameters in vivo in *Prog-Tg* and *LA-Tg* animals in comparison to *Wt* littermate controls. Assessment of echocardiographic and hemodynamic parameters revealed increased end-diastolic septal wall width (EDSW) and end-diastolic posterior wall width normalized to body weight (EDPW), confirming cardiac hypertrophy (Figure 2, D and E, and Supplemental Figure 2, B and C). Cardiac hypertrophy was accompanied by an increase in LV end-diastolic pressure (LVEDP) and a mild increase in LV systolic pressure (LVSP) (Figure 2, F and G) with preserved ejection fraction (Supplemental Table 1). Furthermore, an increased diastolic wall stress, a trend toward increased dP/dt, and a reduced isovolumic relaxation time (IVRT) (Supplemental Table 1 and Supplemental Figure 2, D and E) in *Prog-Tg* mice implicated restrictive filling consistent with a restrictive diastolic function in *Prog-Tg* mice (38). Altogether, our data point toward LV diastolic dysfunction in *Prog-Tg* mice, which is the most prevalent cardiac abnormality observed in HGPS patients (5).

**Figure 3:**
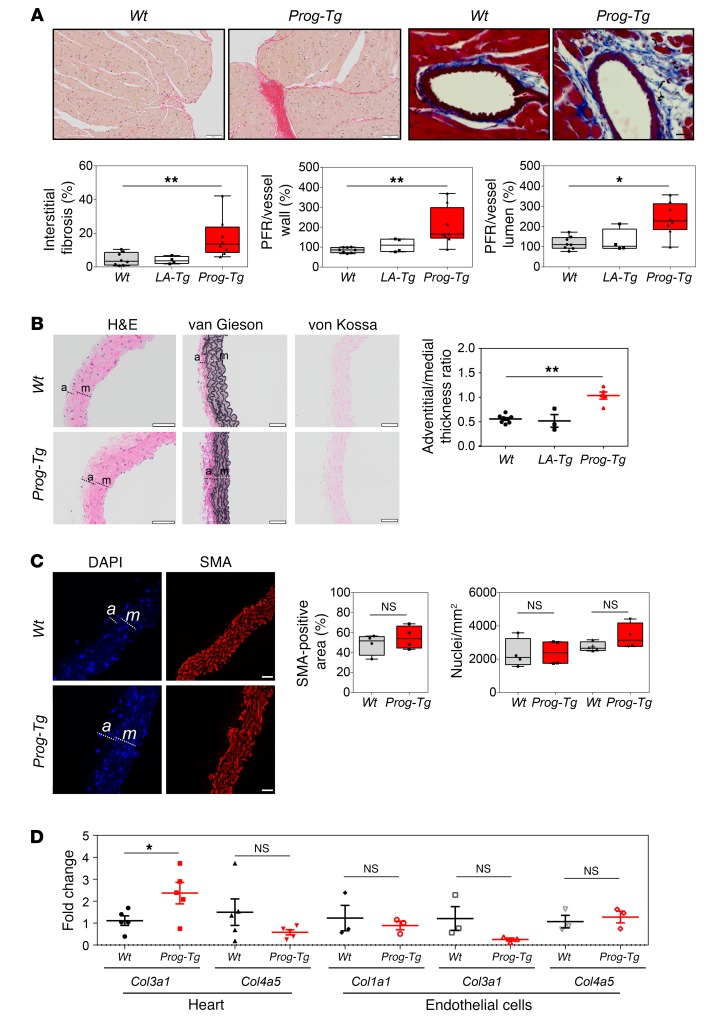
Cardiac fibrosis and adventitial thickening. (**A**) Representative cardiac cross sections stained with picrosirius red (left) and coronary artery sections stained with Masson’s trichrome (right) in *Wt* and *Prog-Tg* mice (representative of littermate pairs analyzed below). Scale bars: 50 μm (left panels) and 20 μm (right panels). Left ventricular myocardial interstitial fibrosis and perivascular fibrosis were evaluated (*n* = 8 *Wt* and *Prog-Tg*, *n* = 4 *LA-Tg*, age >27 weeks). Perivascular fibrosis ratio (PFR) expressed as the ratio of perivascular collagen area to vessel wall area (PFR, vessel wall) and perivascular collagen area to vessel lumen area (PFR, lumen) is shown. Kruskal-Wallis 1-way ANOVA, multiple comparisons versus *Wt* control group reveal *P < 0.05 or **P < 0.01 for *Prog-Tg* and not significant for *LA-Tg* mice (Dunn’s method). (**B**) Representative aorta sections stained with H&E, van Gieson, and von Kossa. Graph shows adventitia (a) to media (m) thickness ratio (*n* = 8 *Wt*, *n* = 3 *LA-Tg*, *n* = 5 *Prog-Tg*; age >30 weeks). Note, only mild elastic distortions and no calcification (von Kossa) in *Prog-Tg* aortas. One-way ANOVA, multiple comparisons versus *Wt* control group reveal **P < 0.01 for *Prog-Tg* and not significant for *LA-Tg* mice (Dunn’s method). (**C**) Representative immunofluorescence images of aortas stained with anti–smooth muscle actin (anti-SMA) and DAPI. Scale bars: 20 μm. Graphs show percentage of SMA-positive area or number of nuclei in adventitia or media (*n* = 4 per genotype; NS, not significant). Mann-Whitney *U* test. (**A** and **C**) Data presented as median (middle line) with boxes encompassing 25th to 75th percentile, and whiskers, minimum to maximum values. (**D**) *Col3a1* (*n* = 5 littermate pairs) and *Col4a5* (*n* = 6 littermate pairs) mRNA levels in heart tissue (age ~25 weeks), and *Col1a1*, *Col3a1*, and *Col4a5* in ECs (*n* = 3, three independent experiments using ECs from 3 littermate pairs). Values were normalized to hypoxanthine phosphoribosyltransferase 1 (*Hprt*) and compared to *Wt* littermate (fold change). *P < 0.05 by paired (littermate tissues) and unpaired (cells) Student’s *t* test. NS, not significant. Data presented as mean ± SEM.

Since increased LV stiffness is a detrimental factor for LV diastolic function (38), we tested for accumulation of fibrous tissue in the hypertrophic myocardium, using picrosirius red and Masson’s trichrome blue staining in heart sections. *Prog-Tg* animals showed significantly increased myocardial interstitial and perivascular fibrosis in comparison with both *Wt* and *LA-Tg* controls (Figure 3A), which is known to impair coronary blood flow and cardiac function (39). Consistent with perivascular fibrosis, we observed an increased adventitial to medial thickness ratio (Figure 3B), indicating adventitial thickening in *Prog-Tg* mice similar to that in HGPS patients (4). In contrast to reports on HGPS patients and mice expressing progerin in all tissues or VSMCs (4, 6), we did not observe loss of VSMCs in *Prog-Tg* mice. Quantification of VSMCs by counting nuclei or by measuring smooth muscle actin–positive (SMA-positive) area in the media showed even a trend toward higher numbers of VSMCs (Figure 3C). Van Gieson staining revealed only subtle elastic tissue distortions, and calcium deposits were absent as tested by von Kossa staining (Figure 3B). Thus, *Prog-Tg* mice did not present severe pathologies in VSMCs, indicating that VSMC loss in HGPS patients is not caused by a dysfunctional endothelium but may be an intrinsic defect of progerin-expressing VSMCs. The impaired, progerin-expressing endothelium, however, causes a strong profibrotic phenotype in *Prog-Tg* mice. To further assess the progerin-mediated fibrotic pathology we analyzed collagen expression patterns in cardiac tissue and ECs. Fibroblast-derived collagen 3 (*Col3a1*) (40) but not endothelium-derived collagen 4 (*Col4a5*) transcript levels were increased in hearts of *Prog-Tg* versus *Wt* littermates (Figure 3D). In ECs, *Col1a1* and *Col4a5* levels were unaltered and *Col3a1* levels were even decreased, indicating that excessive collagen was not primarily derived from the endothelium. Thus, progerin-expressing ECs may mediate a profibrotic response in surrounding tissue. Collectively, transthoracic cardiac echocardiography, histological analyses, and assessment of collagen levels revealed LV hypertrophy and fibrosis of cardiac tissue in *Prog-Tg* mice, similar to the strong fibrotic phenotype in HGPS patients (4) and during vascular aging (14). These findings suggest that the profibrotic CVD pathology in HGPS may be mediated by progerin-expressing endothelium. Importantly, cardiac fibrosis was observed in *Prog-Tg*, but absent in *LA-Tg* mice, suggesting a progerin-specific pathology.

### Downregulation of the antifibrotic eNOS pathway in Prog-Tg ECs.

**Figure 4:**
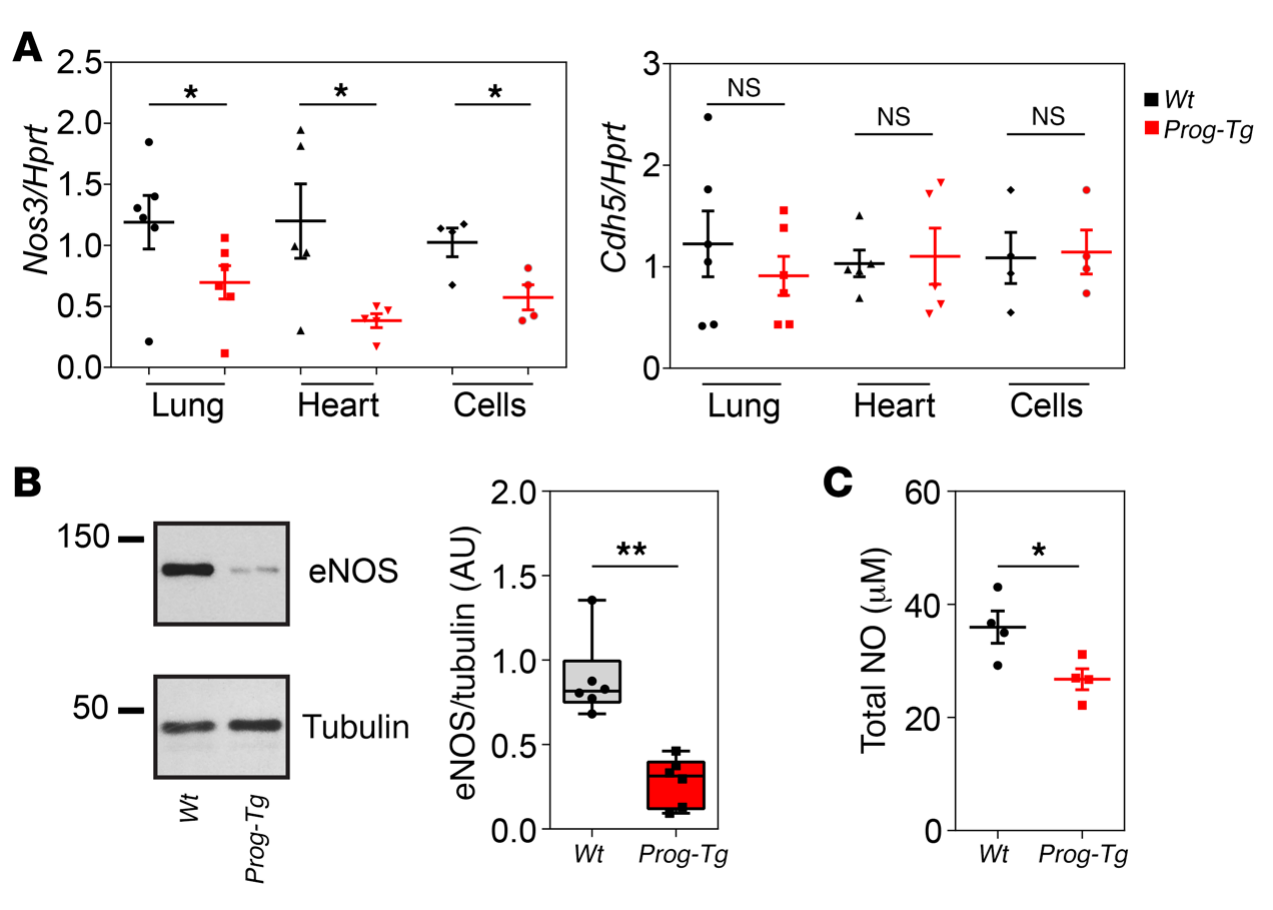
Downregulation of antifibrotic eNOS. (**A**) Expression of *Nos3* and *Cdh5* mRNA in lung, heart, and ECs (*n* = 6, 5, and 4 littermate pairs, respectively; age ~25 weeks). Values were normalized to *Hprt* and compared to those from *Wt* littermate (fold change). *P < 0.05 by paired (littermate tissues) and unpaired (cells) Student’s *t* test. NS, not significant. (**B**) Quantitative immunoblot analysis of lysates from *Wt* and *Prog-Tg* ECs using eNOS antibodies (*n* = 6). **P < 0.01 by Mann-Whitney *U* test. Data presented as median (middle line) with boxes encompassing 25th to 75th percentile, and whiskers, minimum to maximum values. (**C**) Reduction in total nitric oxide in *Prog-Tg* and *Wt* cell extracts. Data are presented in micromolar values of total NOx (nitrite and nitrate) per 2 × 10^5^ cells (*n* = 4 independent experiments). *P < 0.05 by unpaired Student’s *t* test. Data presented as mean ± SEM.

To obtain insight into potential mechanisms underlying how progerin expression in the endothelium contributes to the profibrotic phenotype, we analyzed endothelium-linked pro- and antifibrotic pathways, such as expression of antifibrotic *Nos3* and profibrotic *Icam1* and *Icam2* genes that have been used as prognostic markers for EC dysfunction (21, 22). The antifibrotic effect of endothelium-specific eNOS is mediated through production and secretion of NO that exerts antifibrotic paracrine effects causing downregulation of collagen 3 in surrounding cells (41). *Prog-Tg* mice had significantly reduced levels of *Nos3* transcript in both lung and heart tissue, and eNOS mRNA and protein levels were reduced in *Prog-Tg* ECs, although EC numbers were unaltered (as judged by endothelial marker *Cdh5*; Figure 4, A and B). Accordingly, the total NO metabolite level was significantly reduced in *Prog-Tg* versus *Wt* EC extracts (Figure 4C). To test whether reduced levels of eNOS and NO in *Prog-Tg* ECs affect endothelium-dependent vessel relaxation, we assessed vascular reactivity in isolated aortic segments following administration of endothelium-dependent NO-donor acetylcholine (Ach) or endothelium-independent NO-donor sodium nitroprusside (SNP). Arteries from *Prog-Tg* animals showed a trend toward delayed response to Ach at lower Ach concentration, but not to SNP, with slightly reduced pEC_50_ values (Supplemental Figure 3), indicating a prolonged lag phase in endothelium-dependent vessel relaxation in *Prog-Tg* animals. Therefore, we concluded that the reduced eNOS and NO levels in *Prog-Tg* ECs may be one of the primary causes for the profibrotic response in surrounding tissue but may not be sufficient to significantly impair vascular reactivity. In addition, expression of proinflammatory markers *Icam1* and *Icam2* was not significantly increased in *Prog-Tg* animals (Supplemental Figure 2F).

### ECs in Prog-Tg mice show impaired flow-induced shear stress response and aberrant nucleocytoskeletal connections.

**Figure 5:**
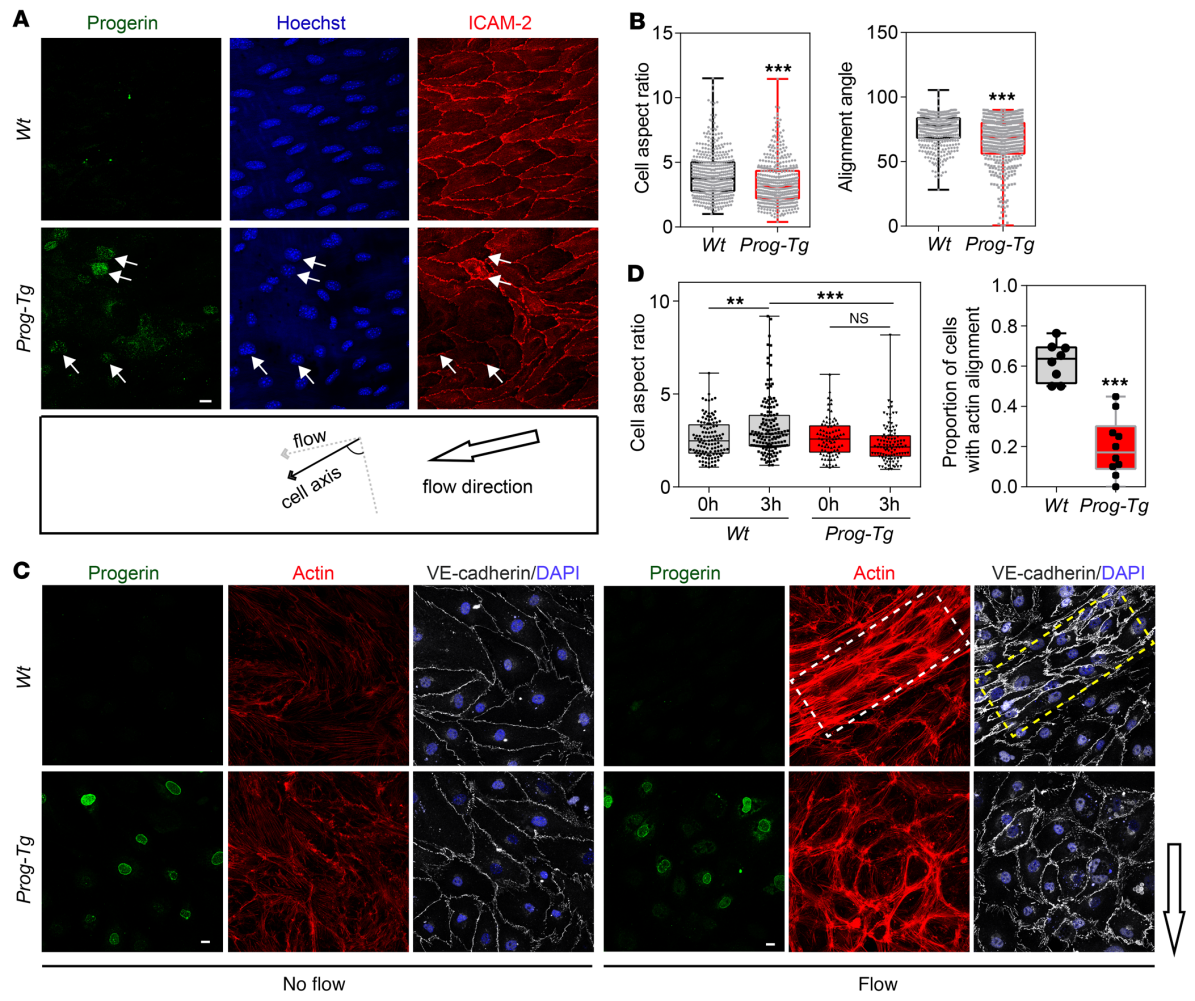
Impaired mechanical response in *Prog-Tg* mice. (**A**) En face view of the endothelial layer in the area of descending aorta isolated from *Wt* and *Prog-Tg* mice and stained with indicated antibodies and Hoechst (DNA) dye. Arrows indicate cells with high progerin levels showing misalignment within aortic sheet (images are representative of *n* = 4 *Wt* and *Prog-Tg* littermate pairs). Scale bar: 10 μm. (**B**) Box plots showing cell aspect ratio and alignment angle (angle between cell major axis and line perpendicular to flow direction) analyzed in equivalent descending aorta regions of *Wt* and *Prog-Tg* littermate pairs (*n* > 400 cells per genotype). ***P < 0.001 by Mann-Whitney *U* test. (**C**) ECs cultured in flow channels before and after exposure to flow of medium (12 dyn/cm^2^) for 3 hours were processed for immunofluorescence microscopy using antibodies and DAPI dye as indicated (images are representative of *n* = 3 independent experiments). Arrow indicates flow direction. Scale bars: 10 μm. (**D**) Box plots showing cell aspect ratios analyzed in *Wt* and *Prog-Tg* ECs after 0-hour (0h) and 3-hour (3h) flow exposure (*n* > 150 cells per condition and per genotype). One-way ANOVA with Dunn’s multiple comparisons test revealed **P < 0.01 (*Wt* 0h versus *Wt* 3h), ***P < 0.001 (*Wt* 3h versus *Prog-Tg* 3h), and not significant (NS, *Prog-Tg* 0h versus 3h and all other comparisons). Right panel, proportion of cells with aligned actin stress fibers in ECs after 3 hours of shear stress exposure. Stress fibers were considered aligned if their direction was parallel to or within 30° to the cell major axis, and misaligned if not (*n* = 4; >250 cells per genotype). ***P < 0.001 by Mann-Whitney *U* test. (**B** and **D**) Data presented as median (middle line) with boxes encompassing 25th to 75th percentile, and whiskers, minimum to maximum values.

**Figure 6:**
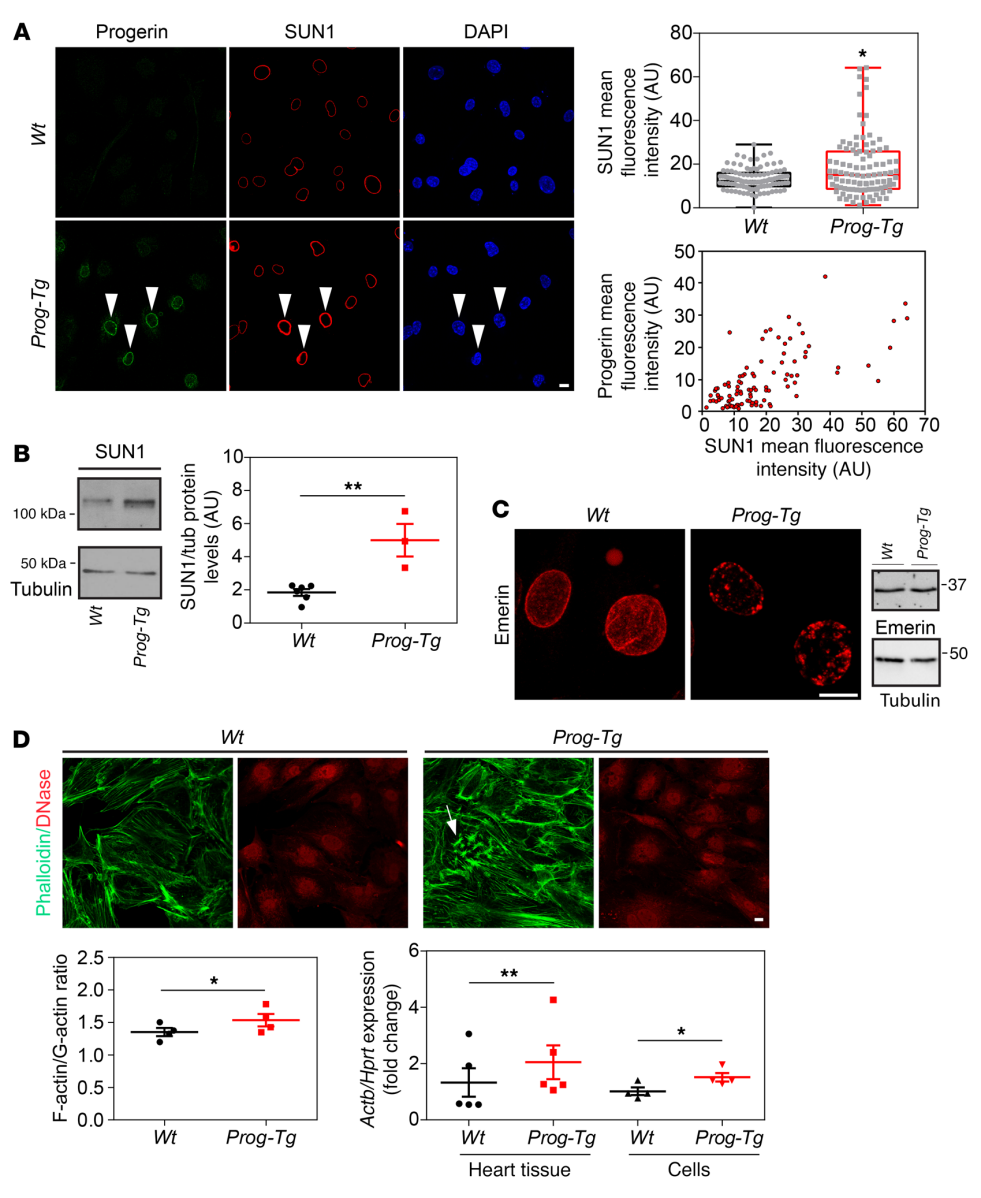
Defective nucleocytoskeletal connections in *Prog-Tg* cells. (**A**) ECs from *Wt* and *Prog-Tg* mice were immunostained with progerin and SUN1 antibodies and DNA dye (DAPI). Arrowheads, cells with high progerin levels show stronger SUN1 staining (images are representative of *n* = 3 independent experiments). Scale bar: 10 μm. Box plots show the distribution of SUN1 mean fluorescence intensities in *Prog-Tg* versus *Wt* ECs (*n* = 100 cells per genotype). Data presented as median (middle line) with boxes encompassing 25th to 75th percentile, and whiskers, minimum to maximum values. *P < 0.05 by Mann-Whitney *U* test. Lower panel, mean progerin fluorescence intensities plotted over mean SUN1 fluorescence intensities in *Prog-Tg* cells. Arbitrary units (AU) of fluorescence, *n* = 101 cells. (**B**) Quantitative immunoblot analysis of *Wt* and *Prog-Tg* endothelial cell lysates (*n* = 6 for *Wt* and *n* = 3 for *Prog-Tg* independent experiments). **P < 0.01. (**C**) Projections of confocal *Z* stacks of *Wt* and *Prog-Tg* ECs stained for emerin. Scale bar: 10 μm. Right panel, immunoblot for emerin and tubulin (loading control, images representative of *n* = 3 independent experiments). (**D**) *Wt* and *Prog-Tg* ECs processed for immunofluorescence microscopy using Atto 488–labeled phalloidin and Texas Red–labeled DNase I, quantitatively evaluated to obtain the F-actin/G-actin ratio (*n* = 4 independent experiments). Arrow, actin “knots” in *Prog-Tg* cells. Scale bar: 10 μm. Lower right, expression levels of actin (*Actb*) in heart tissue (age ~24 weeks, *n* = 5 littermate pairs) and ECs (*n* = 4 independent experiments) from *Prog-Tg* and *Wt* littermates. Values were normalized to *Hprt*. *P < 0.05, **P < 0.01 by paired (littermate tissue) and unpaired (cells) Student’s *t* test (**B** and **D**). Data presented as mean ± SEM.

Next, we investigated potential mechanisms leading to reduced eNOS levels and activation of profibrotic pathways in *Prog-Tg* mice. The endothelium of blood vessels is constantly exposed to blood flow–mediated shear stress, upon which the cells undergo a complex set of cytoskeletal rearrangements and activate antifibrotic, shear stress–responsive molecules such as eNOS (42). We investigated cell alignment in endothelial sheets of descending aorta by immunofluorescence analysis to test potential defects in shear stress response. Progerin-expressing ECs in endothelial sheets were frequently misaligned, as normally observed at curvatures and branches of vessels (43), in contrast to cells in *Wt* aortas that were well aligned in the direction of flow (Figure 5A, arrows). To quantify cell alignment we measured cell aspect ratios and the alignment angle, the angle between the major cell axis and the axis perpendicular to the flow direction (0°, no alignment; 90°, complete alignment) (44). Both, aspect ratios and alignment angles, were significantly lower in *Prog-Tg* versus *Wt* aortic sheets (Figure 5B), confirming misalignment of mutant cells. To test whether the misalignment of ECs in situ was caused by the inability of progerin-expressing cells to respond to flow, primary ECs were exposed to flow shear stress grown in flow channels. Whereas 3-hour flow shear stress exposure induced cell alignment with parallel bundling of actin stress fibers in *Wt* cells, this effect was much less evident in *Prog-Tg* cells, where merely cortical actin increased and nuclei failed to elongate (Figure 5C). A significantly higher proportion of *Wt* cells showed actin alignment (direction of actin stress fibers within 0°–30° to the cell long axis as described in ref. 45) compared with *Prog-Tg* cells after exposure to flow (Figure 5D). Similarly, cell aspect ratios significantly increased in *Wt* but not in *Prog-Tg* ECs after exposure to flow (Figure 5D), indicating a severe impairment in flow shear stress response of progerin-expressing ECs. As direct connections of the actin cytoskeleton to the nuclear envelope play a central role in force transmission upon shear stress (46), we tested the localization and expression levels of SUN1 and -2, members of the LINC complex. Immunofluorescence microscopy and fluorescence intensity measurements (Figure 6A), and immunoblotting (Figure 6B) revealed SUN1 upregulation and accumulation at the nuclear rim of *Prog-Tg* versus *Wt* ECs, as reported also in HGPS fibroblasts (47). Double immunofluorescence staining and quantification revealed that SUN1 protein levels directly correlated with progerin levels (Figure 6A). A similar accumulation was observed for SUN2 (Supplemental Figure 4). Emerin, a nuclear membrane protein involved in nucleocytoskeletal connections (48) and nuclear mechanoresponse (49), was mislocalized in conspicuous patches in *Prog-Tg* but not *Wt* ECs, while emerin protein levels were unaffected (Figure 6C). As emerin promotes actin polymerization (50, 51), we reasoned that changes in emerin may affect actin organization in *Prog-Tg* ECs. Immunofluorescence microscopy of phalloidin-stained cells revealed abundant actin stress fibers in *Prog-Tg* ECs with frequent actin “knots,” suggesting disturbances in actin connections (Figure 6D, arrow). We also found increased F-actin to G-actin ratios in *Prog-Tg* versus *Wt* cells as determined by fluorophore-labeled phalloidin and DNase I staining (Figure 6D). In addition, actin (*Actb*) transcript levels were increased in heart tissue as well as in isolated ECs of *Prog-Tg* animals (Figure 6D). Thus, progerin expression affects the expression and structural organization of actin and impairs nuclear envelope proteins involved in shear stress force transmission.

### Impaired MRTFA mechanosignaling leads to reduction of eNOS levels and activation of profibrotic pathways.

**Figure 7:**
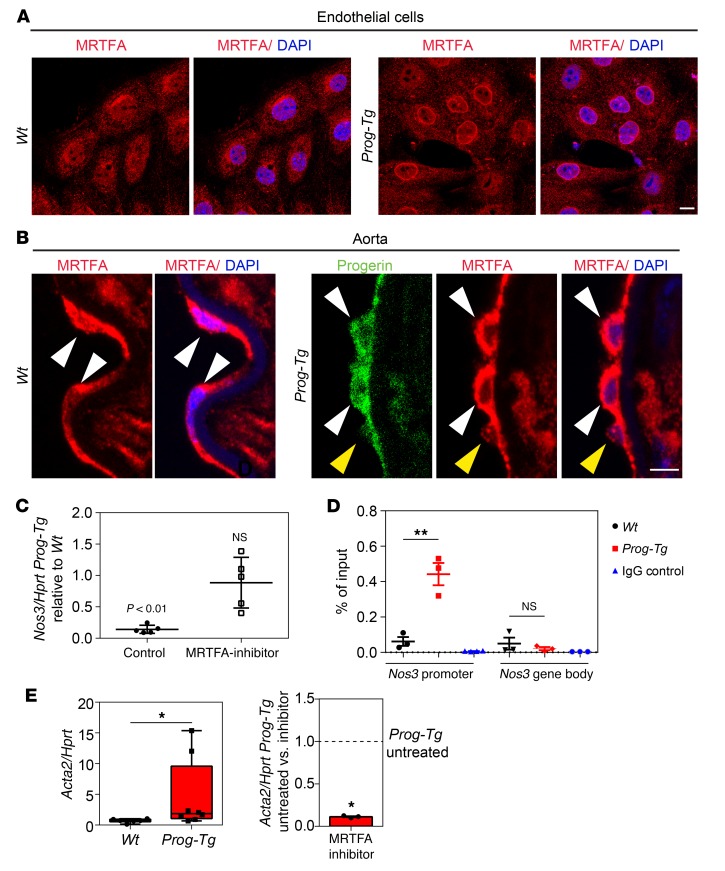
Impaired MRTFA signaling in *Prog-Tg* ECs affects eNOS and profibrotic signaling. (**A**) Immunofluorescence of *Wt* and *Prog-Tg* ECs using MRTFA antibody and DAPI (images are representative of *n* = 3 independent experiments). Scale bar: 10 μm. (**B**) Representative confocal average intensity projections of *Z* stacks from mouse aorta of *Wt* and *Prog-Tg* animals stained with DAPI and MRTFA and progerin antibodies. EC nuclei (arrowheads) lie on internal elastic membrane that displays blue autofluorescence. Arrowheads, MRTFA-positive ECs. Note that MRTFA accumulates at the nuclear periphery of progerin-positive (white arrowheads) but not progerin-negative (yellow arrowheads) ECs (*n* = 3 *Wt* and *Prog-Tg* littermate pairs). Scale bar: 10 μm. (**C**) *Nos3* mRNA in *Wt* and *Prog-Tg* ECs after 24-hour treatment with 15 μM CCG-203971 MRTFA inhibitor or DMSO vehicle control. Values are normalized to *Hprt* and *Nos3*/*Hprt* values in *Prog-Tg* cells are shown relative to values in *Wt* cells after MRTFA-inhibitor and control treatment. *Nos3*/*Hprt* levels in *Wt* cells were arbitrary and set to 1 in both control and CCG-203971 conditions (*n* = 5 independent experiments). *Nos3*/*Hprt* levels in *Prog-Tg* cells relative to *Wt* are not significantly different (NS) after drug treatment, in contrast to control conditions (*P* < 0.01). (**D**) Chromatin immunoprecipitation using anti-MRTFA or goat IgG control. Precipitated DNA was amplified by qPCR with primers spanning *Nos3* promoter or gene body (*n* = 3 independent experiments). **P < 0.01 by unpaired Student’s *t* test (**C** and **D**). Data presented as mean ± SEM. (**E**) *Acta2* levels normalized to *Hprt* in fibroblasts after 3 days of coculture with *Wt* and *Prog-Tg* ECs either left untreated (left) or treated with 25 μM MRTFA inhibitor CCG-203971 (right). Values were subtracted from those obtained in untreated or CCG-203971–treated fibroblast single cultures (*n* = 6 *Wt*, *n* = 8 *Prog-Tg*, and *n* = 3 inhibitor-treated *Prog-Tg* samples). *P < 0.05 by Mann-Whitney *U* test. Data presented as median (middle line) with boxes encompassing 25th to 75th percentile, and whiskers, minimum to maximum values.

Given the observed defects in shear stress mechanosensing in *Prog-Tg* ECs, we sought to identify affected mechanosensitive factors that mediate eNOS expression. The *Nos3* promoter harbors a shear stress–responsive element regulated by mechanosensitive transcription factors such as NF-κB, KLF2, and MRTFA (42, 52). In view of the observed changes in actin and emerin in *Prog-Tg* cells and based on previous reports that MRTFA is regulated by F-actin/G-actin ratios in an emerin- and lamin A–dependent fashion (50), we tested MRTFA localization. MRTFA accumulated at the nuclear periphery of *Prog-Tg* cells, in contrast to the relatively even nuclear and cytosolic distribution in *Wt* cells (Figure 7A). Importantly, MRTFA accumulation at the nuclear periphery was not observed in *LA-Tg* ECs (Supplemental Figure 5). MRTFA redistribution was also found in situ in the endothelial lining of *Prog-Tg* aorta in comparison with the relatively uniform MRTFA distribution in *Wt* aorta (Figure 7B). MRTFA is a transcriptional coactivator promoting cardiac hypertrophy and fibrosis (52–54) and acts as a transcriptional suppressor of *Nos3* (52, 55). To test whether reduced eNOS levels in *Prog-Tg* mice were caused by MRTFA deregulation, we applied MRTFA inhibitor CCG-203971 to *Wt* and *Prog-Tg* ECs for 24 hours. Administration of MRTFA inhibitor rescued eNOS mRNA levels in *Prog-Tg* ECs, while treatment with the vehicle control (DMSO) had no significant effect, indicating that MRTFA is involved in transcriptional repression of eNOS in *Prog-Tg* ECs (Figure 7C). To test if MRTFA downregulates eNOS expression directly by binding to the *Nos3* promoter as previously shown in aortic ECs (56), we performed chromatin immunoprecipitation (ChIP) using an MRTFA antibody and corresponding IgG control followed by quantitative PCR (qPCR) analyses. MRTFA binding to the *Nos3* promoter was significantly increased in *Prog-Tg* versus *Wt* ECs, while MRTFA binding to a region in the *Nos3* gene body was close to background levels (Figure 7D). Thus, MRTFA directly targets the *Nos3* promoter, suppressing eNOS expression in *Prog-Tg* ECs. To test whether the deregulated MRTFA activity also mediates the paracrine profibrotic signaling of *Prog-Tg* ECs, we set up cocultures of ECs with fibroblasts. Primary *Wt* fibroblasts were cultured in the presence of either *Wt* or *Prog-Tg* ECs that were seeded on top in transwell inserts allowing no direct contact except through secretion of extrinsic factors. Profibrotic effects in fibroblasts were tested by assessing expression of SMA (*Acta2*) and collagen 1 (*Col1*) known to be associated with a myofibroblast switch (57). More than 3-fold higher levels of *Acta2* transcripts were found in fibroblasts cocultured in the presence of *Prog-Tg* ECs compared with those cocultured with *Wt* ECs (Figure 7E). Importantly, the profibrotic effect of *Prog-Tg* ECs on fibroblasts was nearly completely abolished in the presence of MRTFA inhibitor (Figure 7E). A similar MRTFA-dependent increase was observed for *Col1* expression, although it did not reach statistical significance (data not shown).

Overall, our results show that progerin expression in ECs leads to defects in nucleocytoskeletal coupling, in flow stress response, and in MRTFA/eNOS signaling, which in turn induce profibrotic changes, cardiovascular stiffening, and cardiac hypertrophy.

## Discussion

Age-related endothelial dysfunction is a prominent initiating event in geriatric CVD and atherosclerosis (13, 17) and is promoted by external insults such as fatty diet, smoking, hypertension, hypercholesterolemia, and increased serum high-sensitivity C-reactive protein, of which only a few are present in patients with HGPS (4, 58). The fact that CVD in HGPS occurs in the absence of most of the classical risk factors suggests that progerin expression directly affects intrinsic properties of the endothelium. So far, vascular pathology in HGPS has been reported to include prominent loss of VSMCs (4, 6, 7), but the contribution of the endothelium to HGPS cardiovascular pathology has not been tested. To address this question we generated a HGPS mouse model with endothelium-restricted progerin expression. Here we show profibrotic changes in the endothelium-specific progerin mice, such as adventitial thickening and increased collagen deposition around coronary arteries, and cardiac interstitial fibrosis, associated with LV hypertrophy and LV diastolic dysfunction. Our data demonstrate that *Prog-Tg* mice phenocopy many aspects of the clinical cardiovascular aberrations in HGPS patients, including the severe profibrotic response and cardiac functional impairment, but they do not present pathologies in the vascular tunica media, such as VSMC loss. Since progerin expression is restricted to the endothelium in *Prog-Tg* mice, the profibrotic cardiovascular pathology in HGPS may be rooted in EC dysfunction, while loss of VSMCs in HGPS is likely caused by intrinsic defects of progerin-expressing VSMCs.

A hallmark of aging-related changes in the endothelium is the reduced secretion of NO, produced by eNOS (20, 59). Endothelium-derived NO is known to mediate endothelium-dependent vasodilation (21) and to exert an antifibrotic paracrine effect on surrounding tissues (41). eNOS and NO levels were significantly reduced in *Prog-Tg* mice and ECs, but *Prog-Tg* aorta showed only a subtle delay in endothelium-dependent relaxation ex vivo. However, other downstream pathways of eNOS/NO, such as the initiation of profibrotic pathways, were clearly affected in *Prog-Tg* mice, as demonstrated by the strong profibrotic response of *Prog-Tg* mice and the profibrotic paracrine signaling of *Prog-Tg* ECs to fibroblasts in cocultures in vitro.

A key feature of healthy atheroprotective blood vessels is their adequate response to shear stress exerted by the blood flow. Thereby a complex set of cytoskeletal rearrangements and nucleocytoskeletal coupling activate atheroprotective and antifibrotic shear stress–responsive pathways such as eNOS (42). Disturbances in flow sensing, which often occurs at branching parts of the arterial tree, impair proper cytoskeletal rearrangements and induce proatherogenic and profibrotic changes including a proinflammatory response, eNOS downregulation, and collagen accumulation. In the present study, we demonstrate a defective cytoskeletal alignment response to shear stress of aortic endothelium and ECs of *Prog-Tg* mice correlating with eNOS downregulation. Our findings are in line with previous reports in other cell systems, showing impaired response of progerin-expressing cells to mechanical stress (60, 61), but, demonstrated for the first time to our knowledge in ECs in vitro and in vivo, our results provide physiological relevance of these defects in HGPS-linked cardiovascular impairment.

Cytoskeletal connections to the nucleus play a central role in the transmission of mechanical forces such as those mediated by shear stress (46, 62) and thus, mutations or impairments in any of the connector molecules, including LINC family members nesprin and SUN1/2 and emerin, were shown to perturb this process (49, 63, 64). We show here severe emerin aggregation and upregulation of SUN1/2 in progerin-expressing ECs, providing a mechanistic explanation for shear stress defects in these cells through aberrant nucleocytoskeletal coupling. In addition, the observed elevated F-actin/G-actin ratio in *Prog-Tg* ECs in conjunction with increased *Actb* transcript levels in tissues and isolated ECs implicates increased cytoskeleton stiffening, expected to hamper the shear stress response. We suggest that actin changes in progerin-expressing cells are rooted in severe aggregation and mislocalization of emerin, which is a key nuclear envelope protein regulating actin polymerization. In line with our hypothesis, recent reports showed that mechanical strain–induced enrichment of emerin at the outer nuclear envelope leads to increased actin polymerization and has a wide range of effects on gene transcription (65). Accordingly, we show aberrant localization of mechanosensitive, F-actin–sensitive, and emerin-regulated transcription factor MRTFA in *Prog-Tg* cells. Importantly, we show that MRTFA inhibition ameliorated the EC-mediated profibrotic paracrine signaling in cocultures, demonstrating a direct involvement of a deregulated MRTFA pathway in the profibrotic effect of progerin-expressing ECs. At a mechanistic level we show that MRTFA binds to the *Nos3* promoter and pharmacological inhibition of MRTFA rescues eNOS levels, suggesting that *Nos3* is a direct MRTFA target gene repressed by MRTFA.

**Figure 8:**
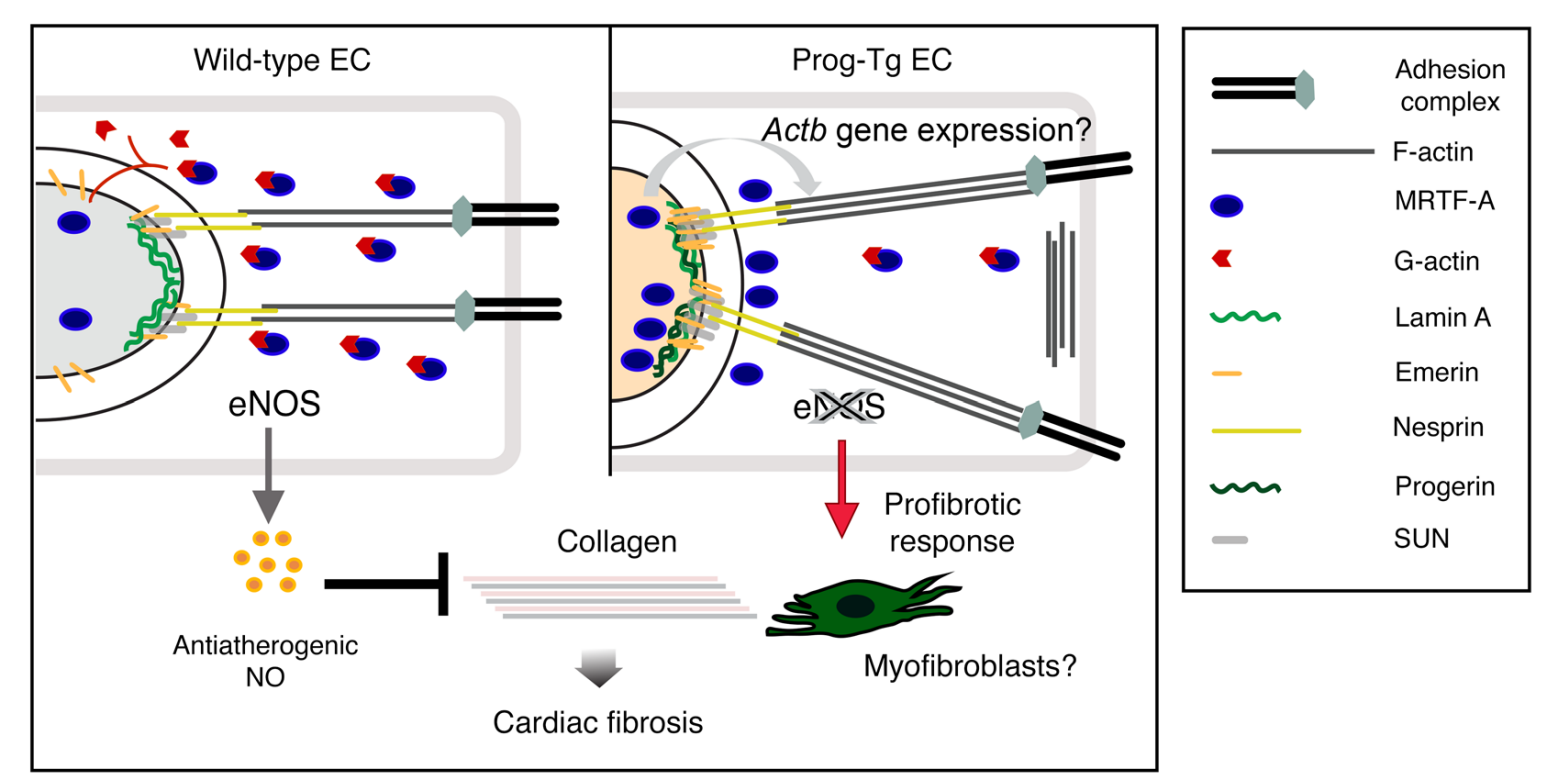
Working model. Progerin accumulation at the nuclear lamina leads to perturbations in F-actin level and actin organization and defects in components involved in nucleocytoskeletal coupling (SUN1/2 and emerin). The defective rigid nucleocytoskeletal links show impaired mechanoresponse and cause MRTFA accumulation at the nuclear periphery. MRTFA or associated complexes exert presumably a positive feedback loop on *Actb* expression (looped arrow). These changes set off the profibrotic signaling cascade (indicated by pale orange color of the nucleus), such as downregulation of eNOS and reduced secretion of NO. Absence of endothelium-derived atheroprotective NO and presumably other unidentified factors leads to increased collagen production in other cell types as shown for fibroblasts through a switch to myofibroblasts, leading to collagen deposition and cardiac fibrosis.

We propose a model (Figure 8) in which progerin accumulation at the nuclear lamina causes upregulation of SUN1/2, emerin aggregation, and F-actin increase, leading to the formation of more rigid nucleocytoskeletal links. Since MRTFA is involved in transcriptional regulation of many cytoskeletal genes (66), including SMA (*Acta2*), it is conceivable that it also exerts a positive feed-back regulation on actin expression. In addition, the MRTFA-mediated downregulation of atheroprotective eNOS results in a decrease of NO secretion by ECs, promoting fibrosis in other, non-endothelial, cell types. In conclusion, we show for the first time to our knowledge that progerin expression in endothelial tissue drives profibrotic changes leading to cardiovascular defects and premature death in mice. Mechanistically, we provide the MRTFA/eNOS axis–rooted mechanosensing defects as the basis for profibrotic changes and vascular insults in HGPS. Our results open new possible intervention strategies for the treatment of progeria and, in view of shear stress insensitivity occurring during normal endothelial aging (20, 23, 67) and of progerin accumulation in arteries of aged individuals (4), our study will also help to elucidate molecular processes of CVD in normal aging and find ways to unwrinkle the aged arteries.

## Methods

### Generation of bitransgenic mice.

Bitransgenic *Prog-Tg* and *LA-Tg* mice were generated by crossing tet operon–driven transgenic mice (C57BL/6J background) carrying either the wild-type (*tetop-LA^Wt^*) or HGPS mutant (1824C>T; G608G; *tetop-LA^G608G^*) lamin A minigene (32) with transgenic mice expressing a tetracycline-responsive (TA-responsive) transcriptional activator under the control of the EC-specific VE-cadherin promoter (*Cdh5-tTA* mice, Jackson Laboratories MGI:4437711, *FVB* background, originally generated as described in ref. 34). All experiments were performed with bitransgenic mice backcrossed onto the C57BL/6J background for 4 generations and their corresponding single-transgenic *tetop-LA^wt^* and *tetop-LA^G608G^* littermate mice (N4 generation; ~94%). Mice were kept in the absence of doxycycline, allowing constitutive expression of the corresponding transgenes in bitransgenic animals. For genotyping, DNA was extracted from mouse distal phalanx. The following primers were used: DNA lamin A minigene, forward 5′-GCAACAAGTCCAATGAGGACCA-3′ and reverse 5′-GTCCCAGATTACATGATGC-3′; DNA *Cdh5-*tTA, forward 5′-CGCTGTGGGGCATTTTACTTTAG-3′ and reverse 5′-CATGTCCAGATCGAAATCGTC-3′. For weight and survival curve analysis the indicated number of littermate pairs was monitored for 35 or 40 weeks.

### Cells.

In all experiments ECs refer to primary ECs isolated from lung (see below), used at a maximum passage number of 3. Primary HGPS patient–derived dermal fibroblast cell line HGADFN003 (2 yr, 10 population doublings [PDs]) was obtained from the Progeria Research Foundation. For assessing EC-specific progerin expression in Figure 1A, a mixed cell population isolated from lungs of *Prog-Tg* mice was depleted of ECs (non-EC) by magnetic bead separation (68). Primary fibroblasts were isolated from lungs of newborn *Wt* (C57BL/6J background) mice by collagenase I treatment (69).

### EC isolation and culture.

Primary ECs were prepared from lungs of *Wt*, *LA-Tg*, and *Prog-Tg* newborn mice following a protocol adapted from Dong et al. (70). Minced lungs were treated with 200 U/ml collagenase I (MilliporeSigma) for 45 minutes at 37°C and passed through a 19-gauge syringe and a 70-μm cell strainer. After centrifugation, cells were seeded on gelatin-coated (1% w/v) and fibronectin-coated (1 μg/ml) plates in DMEM supplemented with 20% FCS, EC growth supplement (Alfa Aesar, BT-203), 25 mM HEPES, 50 U/ml penicillin, 50 μg/ml streptomycin, 2 mM L-glutamine, 1 mM nonessential amino acids, 1 mM sodium pyruvate, and 139 μg/ml heparin (complete culture medium). ECs were grown to confluence for 3 or 4 days, detached with trypsin, and isolated by magnetic bead sorting using rat anti-ICAM2 antibodies (BD Biosciences Pharmigen, clone 3C4 [mlC2/4], 553326) coupled to sheep anti–rat IgG paramagnetic Dyna-beads (Invitrogen, 11035). For some experiments, sorting was repeated after 3 or 4 days. For collecting EC-depleted cell populations, supernatants of the first magnetic bead separation were seeded on plastic dishes (non-ECs).

### Cocultures.

ECs (maximum passage number 3) were plated in the inserts, and primary fibroblasts (maximum passage 2) at the bottom of 12-well Transwell plates (Costar Corning Incorporated, 3460; pore size 0.4 μm) separately at a density of 3.6 × 10^4^ cells/cm^2^ and 2.4 × 10^4^ cells/cm^2^, respectively, and allowed to adhere overnight. After 24 hours, the 2 cell types were placed in coculture by transferring the inserts covered with ECs into wells containing the fibroblasts. On the second day, fibroblasts were either mock treated with DMSO or treated with the MRTFA inhibitor CCG-203971 (Tocris Bioscience). After 3 days of coculture, fibroblast RNA was isolated and qPCR performed. Profibrotic effects were tested by expression of SMA (*Acta2*) and collagen 1 (*Col1a1*). Obtained values were subtracted from values for untreated or CCG-203971 inhibitor-treated fibroblast-only cultures.

### Immunoblotting and antibodies.

For direct lysis, confluent EC cultures were washed twice with PBS, and overlaid with 50 mM Tris-HCl, pH 6.8, 100 mM DTT, 2% SDS, 0.1% bromphenol blue, and 10% glycerol. Lysates were separated by SDS-PAGE and transferred onto nitrocellulose membranes. Membranes were blocked and incubated with primary antibodies as described previously (69). For protein detection, membranes were incubated with peroxidase-coupled secondary antibodies (Jackson Laboratories) for 1 hour and processed using the Amersham ECL Prime Western Blotting Detection Reagent. Protein bands were quantified using ChemiDoc MP Imaging System with Image Lab Software. The following primary antibodies were used: mouse monoclonal antibody (mAb) against lamin A/C (Santa Cruz Biotechnology, clone E-1, sc-376248), mAb against human lamin A+C (Chemicon, clone JoL2, mab3211, Abcam), goat anti-MRTFA (Santa Cruz Biotechnology, sc-21558), mAb against progerin (Alexis Biochemicals, clone 13A4, provided by Egon Ogris, Medical University Vienna, Vienna, Austria), mAb against α-tubulin (MilliporeSigma, clone B-5-1-2, T5168), mAb against emerin (Leica Biosystems, clone 4G5, NCL-emerin), rabbit anti-emerin (for immunoblotting, Atlas Antibodies, HPA000609), goat anti–VE-cadherin antibodies (Santa Cruz Biotechnology, sc-6458), rabbit anti-PECAM1 (LSBio, LS-B4737), mAb against eNOS (BD Biosciences, 610297), rabbit affinity-purified antibodies against actin (MilliporeSigma, A2066), guinea pig anti-SUN1 and -SUN2 antibodies (a gift from Manfred Alsheimer, University of Würzburg, Würzburg, Germany). See complete unedited blots in the supplemental material.

### Histology.

Mice (6–8 months old) were euthanized with isoflurane. Tissues were fixed in 4% paraformaldehyde (PFA), embedded in paraffin, and sectioned with a microtome (Leica) at 5 μm. For aortic tissue, PFA-fixed samples were incubated overnight in 20% sucrose in PBS, embedded in OCT (Tissue-Tek), rapidly frozen, and cryosectioned (1 μm thick). Coronal sections at the equator were processed for H&E and fluorescein-labeled wheat germ agglutinin (Vector Laboratories, FL-1021) staining, and Masson’s trichrome/or picrosirius red staining (71) to assess cardiomyocyte cross-sectional area (CSA) and cardiac fibrosis, respectively. For CSA assessment, only round-to-ovoid cells with visible round nuclei from randomly chosen images were analyzed. Samples were examined using a Zeiss Axiophot microscope equipped with an AxioCam camera. For assessment of interstitial fibrosis, the picrosirius red–positive area was determined using the color thresholding method in ImageJ software (NIH). LV interstitial fibrosis was calculated as the sum of all picrosirius red–positive areas divided by the total section surface, in at least 6 images per mouse. Perivascular fibrosis was evaluated as the ratio of perivascular collagen area to vessel lumen area (72) or to vessel wall area (39). Ratios were averaged from images of arteries of more than 10 sections per mouse. All histological evaluations were performed in a blinded manner.

### Immunofluorescence microscopy and image analysis.

Cells grown in ibidi chambers were fixed in 4% PFA for 10 minutes, permeabilized in 0.5% Triton X-100 in PBS for 5 minutes, and blocked in 3% BSA in PBS. Cells were incubated with primary antibodies (see above) in blocking buffer for 1 hour at room temperature, or in the case of goat antibodies against MRTFA at 4°C overnight. Cells were washed 3 times with PBS and probed with fluorescently labeled secondary antibodies (Thermo Fisher Scientific, DyLight Fluor secondary antibodies) in PBS for 1 hour at room temperature. All samples were counterstained with Hoechst or DAPI (1:10,000 in PBS) for 5 minutes at room temperature and mounted in ibidi Mounting Medium (catalog 50001). For immunofluorescence staining on paraffin-embedded tissue sections, antigen retrieval using a pressure cooker was performed (32) with subsequent staining using the MOM Basic Kit (Vector Laboratories). Images were acquired on a confocal laser scanning microscope (LSM 710, Carl Zeiss) using 100×/1.46 alpha, 63×/1.4, and 40×/1.4 Plan-Apochromat oil immersion objectives. Images were processed in ImageJ and Adobe Photoshop CS4 software. For characterization of cell alignment, the cell aspect ratio, alignment angle, and actin stress fiber alignment were calculated as described (44, 45). Briefly, aspect ratio was determined by dividing the cell long axis by the cell short axis, and alignment angle was measured as the angle between the major cell axis and the axis perpendicular to flow direction using ImageJ software. For actin stress fiber alignment, the proportion of cells with aligned stress fibers after exposure to 3-hour shear stress was determined from analysis of 10 different images obtained from 4 biological replicates of *Wt* and *Prog-Tg* ECs. Stress fibers were considered aligned if their direction was within 0°–30° between the major cell and flow axis. For quantification of Atto 488–phalloidin and Texas Red–DNase fluorescence intensities, images were captured using identical exposure times for *Wt* and *Prog-Tg* cells and unprocessed raw data were used. Digital images for production of printable figures were adjusted for brightness and contrast equally for *Wt* and *Prog-Tg* cells and exported to Adobe Illustrator.

### F-actin/G-actin assay.

Cells were processed as for immunofluorescence except that 4% PFA was prepared in cytoskeleton buffer (10 mM MES, 3 mM MgCl_2_, 138 mM KCl, 2 mM EGTA, 320 mM sucrose, pH 6.1) (73). For G-actin staining we used Alexa Fluor 594 Deoxyribonuclease I conjugate (Molecular Probes) and for F-actin phalloidin–Atto 488 (MilliporeSigma).

### Shear stress assay and en face aortic preparations.

Cells (1.5 × 10^4^) were grown on 0.2% gelatin– and 1 μg/ml fibronectin–coated flow chambers (80601, ibidi) and left to adhere for approximately 24 hours. Cells were exposed for 3 hours to flow generating a shear stress of 12 dynes/cm^2^ (equivalent to flow in human arteries) (74). En face aorta preparations were performed as described previously (68). Briefly, animals were sacrificed via isoflurane inhalation, the chest cavity was cut open, the aorta exposed and washed free of blood by perfusing in situ with 2 mM EDTA in PBS, with the right atrium open to allow free flow. The aorta was dissected free from surrounding tissue and slit opened longitudinally. The aorta was pinned flat (with the intima surface up) on a silicone cell culture dish and processed for immunofluorescence staining.

### RNA isolation and gene expression analysis.

Heart and lung tissues were disrupted and homogenized using ceramic beads in RLT buffer (Qiagen Mini Kit) in a Precellys 24 tissue homogenizer according to the manufacturer’s protocols (Bertin Instruments). RNA from tissues and cells was isolated using the RNeasy Mini Kit (Qiagen), and total RNA was quantified by a spectrophotometer (NanoDrop Technologies, ND-1000). cDNA was generated using RevertAid reverse transcriptase (Thermo Fisher Scientific). qPCR was performed using the primers listed in Supplemental Table 2. All reactions were done in triplicate on an Eppendorf RealPlex 2 Mastercycler with KAPA SYBR Green PCR master mix (Peqlab) according to the manufacturer’s instructions. Results were normalized to expression of *Hprt* and presented as fold increase relative to *Wt* littermate animals based on the ΔΔCt method.

### NO measurement.

Cells plated in 12-well plates were lysed in lysis buffer (0.5% Triton X-100, 20 mM Tris-HCl, pH 7.2, 137 mM NaCl, 10% glycerol, 5 mM EDTA) supplemented with a protease inhibitor cocktail tablet (Roche), sheared with a 27-gauge needle, and cleared at 16,000 *g* for 15 minutes. Total NO content was determined by measuring nitrite with Griess reagent (Arbor Assays). Briefly, samples were diluted 1:3 in assay buffer (Arbor Assays) and filtered through spin filters (10 kDa cutoff; Corning Spin-X UF 500, 431478) to remove protein. In order to assay for total NO, nitrate was first converted to nitrite with NADH reductase followed by addition of color reagents, sulfanilamide, and *n*-(1-naphthyl)ethylenediamine. Absorbance was measured at 550 nm using a microplate reader. Nitrite concentration was determined using nitrite standards provided by the manufacturer.

### ChIP.

ChIP was performed as previously described (75) with slight modifications. Briefly, chromatin was cross-linked with 1% formaldehyde, cells were incubated in lysis buffer (1% SDS, 0.01 M EDTA, 0.05 M Tris pH 8.1, 0.1 mM PMSF in isopropanol) and DNA was fragmented into approximately 500-bp fragments using a Bioruptor (Diagenode Pico Bioruptor). Aliquots of lysates containing 13 μg chromatin were used for immunoprecipitation using anti-MRTFA (Santa Cruz Biotechnology, sc-21558) or goat IgG (76). Cross-links were reversed by heating at 65°C in the presence of 0.2 M NaCl and DNA fragments were recovered using the ChIP DNA Clean and Concentration Kit from Zymo Research (catalog D5205). Primers for the promoter region (Supplemental Table 2) were located within 400 bp upstream of the transcription start site (52).

### Transthoracic echocardiography.

Echocardiography was performed using a Vevo 2100 Imaging System with a 40-MHz linear probe (Visualsonics). The animals were anesthetized by 1%–1.5% isoflurane. ECG monitoring was obtained via limb electrodes. For the LV function and dimension analysis, parasternal long-axis view and short-axis view were obtained. For each parameter, a mean of 3 cardiac cycles in each view was used. Doppler mitral flow profiles were acquired using pulsed-wave Doppler in the apical 4-chamber view. Attention was paid to place the sample volume at the mitral leaflets tip parallel to the blood to record the maximal flow velocities. Isovolumic relaxation time (IVRT) measurement was performed by simultaneously recording pulsed-wave Doppler of the mitral and aortic flow. IVRT was measured as the time interval between aortic valve closure and mitral valve opening. E′ was obtained by tissue Doppler in a 4-chamber view by placing the velocity sample at the septal mitral annulus. E′ indicated the peak mitral annular velocity during early filling.

### Assessment of LV hemodynamic function in vivo.

Mice were anesthetized with a mixture of ketamine and xylazine and analgesic therapy was applied subcutaneously with buprenorphine 0.12 mg/kg body weight. The thorax was opened and a microtip catheter (SPR-1000, Millar Instruments) was inserted into LV chamber. Hemodynamic parameters such as LVSP, LVEDP, heart rate (HR), and LV contractility performance (dP/dt) were continuously registered in a Powerlab 8/30 system with LabChart (v7.3.2) software (both ADInstruments). Systolic and diastolic wall stress were computed accordingly: LVP × LV radius/(2 × wall thickness) (77). The LV radius and LV wall thickness were assessed by echocardiography.

### Assessment of vascular reactivity in isolated aortic rings.

The thoracic aorta was dissected and placed in cold physiological buffer: 119 mM NaCl, 4.7 mM KCl, 2.5 mM CaCl_2_ × 2H_2_O, 1.17 mM MgSO_4_ × 7H_2_O, 20 mM NaHCO_3_, 1.18 mM KH_2_PO_4_, 0.027 mM EDTA, 10.5 mM glucose; bubbled with a mixture of 95% O_2_ and 5% CO_2_, resulting in pH 7.4. Aortic rings (2–3 mm) were mounted onto a multichamber isometric myograph system (Model 620M, Danish Myo Technology) and equilibrated at 37°C. The resting tension was reached by the normalization process using the ADInstuments Normalization module, where the segments of aorta were normalized to their length and diameter. At the beginning of each experiment, the ability of the preparation to develop a contraction was assessed by exposing the aorta segments to a high KCl solution (124 mM). Endothelium-dependent relaxations were determined by administration of cumulatively increasing concentrations of acetylcholine (10^–9^ to 10^–5^ M, MilliporeSigma) to aortas precontracted with phenylephrine (10^–10^ to 10^–6^ M, MilliporeSigma). The aorta segments were then washed 3 times every 10 minutes for 30 minutes. The endothelium-independent relaxation was tested by sodium nitroprusside (SNP, 0.1 nM to 10 μM, Merck), following precontraction with phenylephrine (10 μM, MilliporeSigma). Data were recorded and analyzed with the LabChart Pro evaluation program (ADInstruments).

### Statistics.

Data are presented as the mean ± SEM or median with minimum and maximum values (whiskers). All experiments using *Wt* and *Prog-Tg* ECs were performed at least 3 times using different primary cells isolated from corresponding littermate pairs. Statistical analyses were performed using either SigmaPlot software or GraphPad Prism statistical software. Comparisons of longitudinal data for growth curve analysis were done with 2-way repeated-measures analysis of variance (ANOVA) with subsequent Holm-Sidak post hoc test. If 3 independent groups were evaluated, data were analyzed using 1-way ANOVA. Comparison of 2 groups was made by unpaired Student’s 2-tailed *t* test. A paired *t* test was used in cases of experiments with littermate pairs. For evaluations of non-normally distributed data, a nonparametric Mann-Whitney rank-sum test was used. Data were considered statistically significantly different if *P* < 0.05. For comparisons among multiple groups, we used a 1-way ANOVA with Dunn’s multiple comparisons test, except where indicated otherwise. Curves in Supplemental Figure 3 were generated using a nonlinear fit model in GraphPad Prism.

### Study approval.

All mouse experiments were approved by the regional Ethics Committee for Laboratory Animal Experiments at the Medical University of Vienna and the Austrian Ministry of Science Research and Economy (66.009/0321-WF/V/3b/2016), according to Austrian Law BGBI. I Nr.114/2012 (TVG2012) and in accordance with the Guide for the Care and Use of Laboratory Animals published by the US National Institutes of Health (NIH Publication No. 85-23, revised 1996).

## Author contributions

SOM conceived of, performed, and analyzed experiments, prepared figures, and cowrote the manuscript. RF conceived of experiments and cowrote the manuscript. OH, AK, and BKP provided advice on in vivo cardiac data. OH and AK performed and evaluated transthoracic echocardiography and left ventricular pressure characterization, and PLS and AK performed ex vivo vessel reactivity assays, within the group of BKP. All above listed authors edited the manuscript. CM, FS, IF, and PF performed experiments. ME generated and tested mice carrying lamin A minigenes.

## Supplementary Material

Supplemental data

## Version 1 11/13/2018

In-Press Preview

## Version 2 12/18/2018

Electronic publication

## Version 3 02/01/2019

Print issue publication
