# Supplementary material for: Endothelial progerin expression causes cardiovascular pathology through an impaired mechanoresponse
Source: J Clin Invest. 2018 Dec 18;129(2):531–45. doi: 10.1172/JCI121297 (PMC6355303; doi:10.1172/JCI121297)
Supplement: Supplemental data [file jci-129-121297-s031.pdf]

SUPPLEMENTAL DATA, Osmanagic-Myers et al.

1. Supplemental Figures and Figure Legends

2. Supplemental Tables

Supplemental Figure 1. Endothelium-specific expression of human lamin A and progerin in *Prog-Tg* mice (related to Figure 1).

Supplemental Figure 2. Analysis of hypertrophy and inflammatory markers and representative echocardiography measurements in the heart. (related to Figure 2).

Supplemental Figure 3. Vascular reactivity in isolated aortic segments of *Prog-Tg* mice. (related to Figure 4).

Supplemental Figure 4. Accumulation of SUN2 in *Prog-Tg* endothelial cells. (related to Figure 6).

Supplemental Figure 5. MRTFA localization is unaltered in *LA-Tg* endothelial cells. (related to Figure 7).

Supplemental Table 1. Hemodynamic and echocardiographic parameters. (related to Fig. 2)

Supplemental Table 2. Primers used for quantitative real-time PCR analysis.

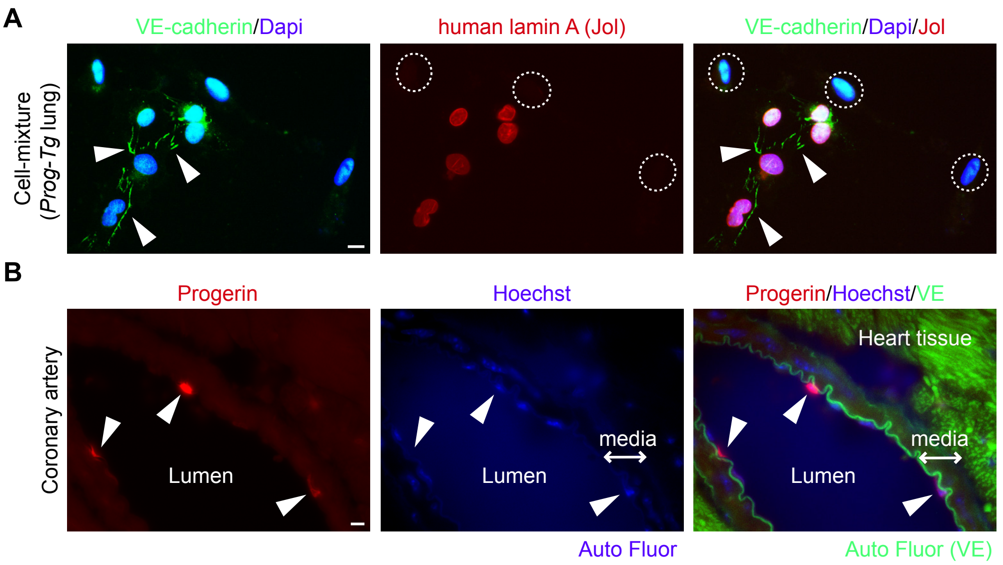

Supplemental Figure 1. Endothelium-specific expression of human lamin A and progerin in *Prog-Tg* mice. (A) Immunofluorescence microscopy of a mixture of cell types obtained from lung tissue using antibodies to human lamin A (Jol) and to endothelial marker VE-cadherin. Arrowheads, VE-cadherin marked endothelial-specific cell-cell junctions. Encircled cells, non-endothelial cells (absence of VE-cadherin cell-cell junctions). Note that only cells positively stained for VE-cadherin junction marker were detected positive for human lamin A. Scale bar, 10 µm.

(B) Immunofluorescence image of larger area from coronary artery (compared to Figure 1C) showing surrounding cardiac tissue from *Prog-Tg* animals stained with progerin antibody, VE-cadherin antibody and DNA dye Hoechst. Progerin expression is confined to intimal layer (arrowheads) and absent in surrounding area. Hoechst and green autofluorescence signals are used to identify internal- and external elastic lamina (IEL) to mark boundaries of the medial layer (media, double arrow). Note, that the VE-cadherin marked intimal layer signal merges with the IEL autofluorescence signal. Scale bar, 10 µm. (A and B, representative of n=3 independent experiments).

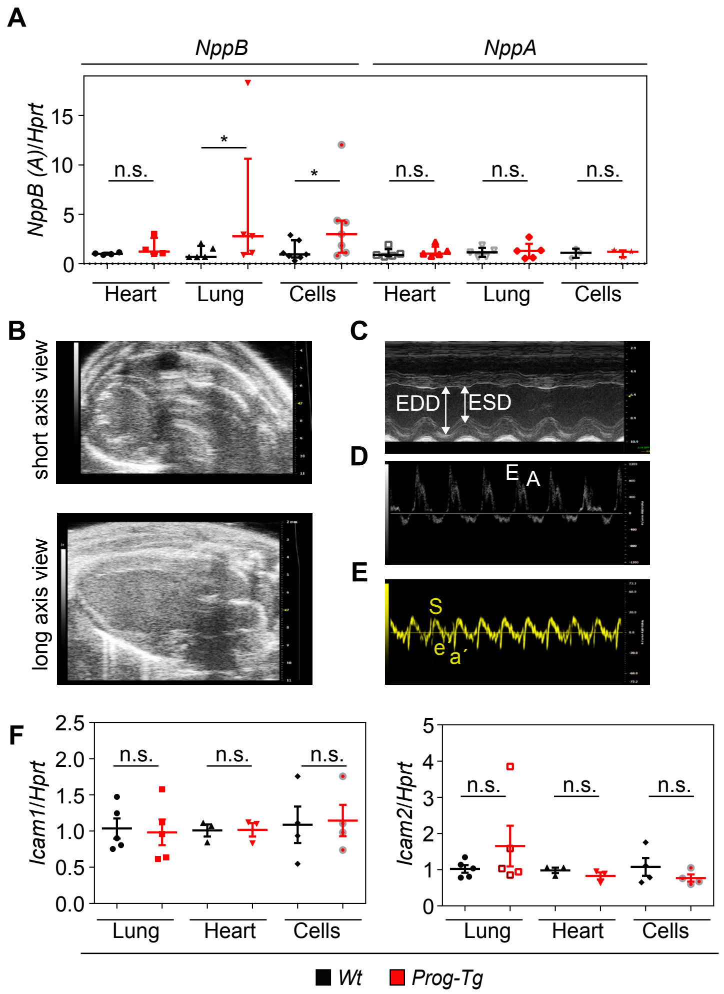

Supplemental Figure 2. Analysis of hypertrophy and inflammatory markers and representative echocardiography measurements in the heart. (A) Expression of *NppB* and *NppA* in heart and lung tissue of *Prog-Tg* animals at ~25 weeks (n=5 littermate pairs) and in *Prog-Tg* ECs (n=7) normalized to hypoxanthine phosphoribosyl-transferase 1 (*Hprt*) and compared to those from *Wt* littermates (fold change) and *Wt* ECs, respectively. n.s. not significant, *p<0.05. Wilcoxon signed rank test (littermate tissues) and Mann-Whitney U test (cells). Median (middle line) and whiskers, 25th and 75th percentile. **(B)** Representative echocardiography 2D mode with a short and long axis view of the left ventricle allowing to obtain a M Mode tracing of the left ventricle in **(C)** in order to evaluate the left ventricle EF, diameters and wall thickness. **(D)** Representative mitral Doppler flow profile for measurement of IVRT and the combined parameter E/e'. **(E)** Representative tissue Doppler tracing of the septum at the mitral annulus level. (B-E) representative of n=5 littermate pairs. **(F)** Gene expression of *Icam1* and *Icam2* in lung, heart and isolated lung endothelial cell cultures (n= 5 and 3 littermate pairs, n=4 independent experiments for cells). All mice were at the age of ~25 weeks. Values normalized to *Hprt* were compared to those from *Wt* littermate or *Wt* cells (fold change). n.s. not significant. Paired (littermate tissues) and unpaired (cells) Student's t-test. Mean and error bars indicate SEM.

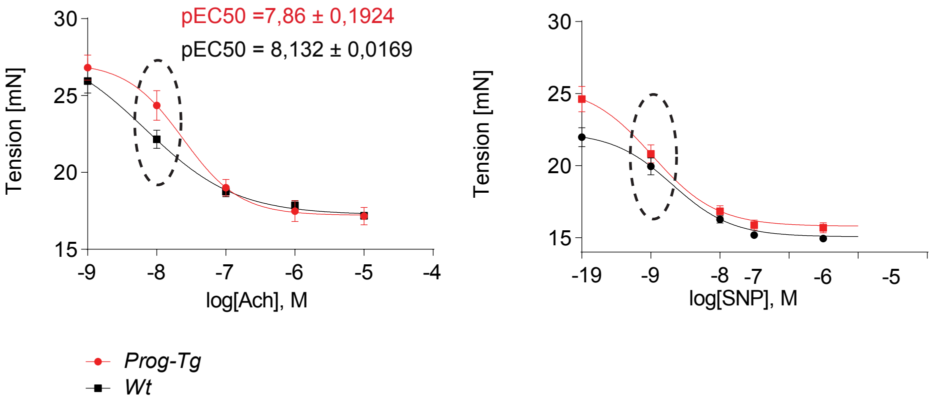

Supplemental Figure 3. Vascular reactivity in isolated aortic segments of *Prog-Tg* mice. Aortic segments were isolated from mice and were mounted onto a Multi Wire Myograph System (DMT 620 M) for determination of endothelium-dependent relaxation and endothelium-independent relaxation by cumulatively increasing concentrations of acetylcholine (Ach, 10^{-9} to 10^{-5} mol/l) and sodium nitroprusside (SNP, 10^{-10} to 10^{-6} mol/l), respectively. Tension in (mN) and log (concentration) in (mol/l) is shown (n=4 animals per genotype; age>30 weeks). Note that in *Prog-Tg* versus *Wt* aortic segments a tendentiously prolonged lag phase and reduced pEC50 values (p=0.2) were observed only for endothelium-dependent relaxation. Error bars denote mean ± SEM.

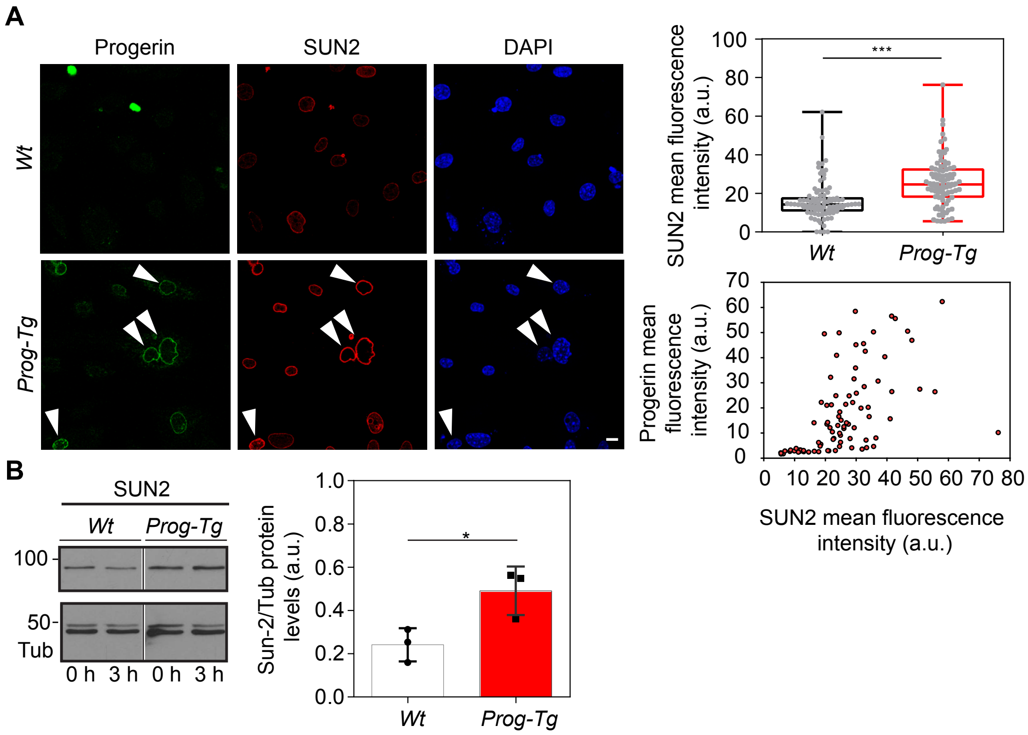

Supplemental Figure 4. Accumulation of SUN2 in *Prog-Tg* endothelial cells. (A) Representative image of endothelial cells from *Wt* and *Prog-Tg* mice immunostained with SUN2 antibody (representative of n=3 independent experiments). Scale bar, 10 µm. Box plots show the distribution of SUN2 mean fluorescence intensities in *Prog-Tg* and *Wt* endothelial cells (n=100 cells per genotype). *** p<0.001, Mann-Whitney U test. Median (middle line) with boxes encompassing 25th to 75th percentile and whiskers, minimum to maximum values Lower panel, mean progerin fluorescence intensities plotted over mean SUN2 fluorescence intensities in *Prog-Tg* cells (n=87 cells). Arbitrary units (a.u.) of fluorescence are shown. (B) Quantitative immunoblot analysis of lysates (0 h, untreated) from *Wt* and *Prog-Tg* endothelial cells (*Wt* and *Prog-Tg* samples from different parts of the same gel; n=3), * p<0.05, unpaired Student's t-test. Mean and error bars indicate SEM. 3h, flow exposed samples.

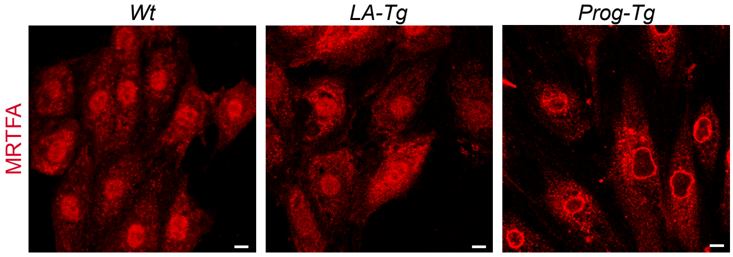

Supplemental Figure 5. MRTFA localization is unaltered in *LA-Tg* endothelial cells. Endothelial cells isolated from *Wt*, *LA-Tg* and *Prog-Tg* mice were processed for immunofluorescence microscopy using MRTFA antibody (representative of n=3 independent experiments). Scale bars, 10 μm.

Supplemental Table 1. Hemodynamic and echocardiographic parameters.

**Hemodynamic parameters**	**LM_*Prog-Tg***	***Prog-Tg***	**LM_*LA-Tg***	***LA-Tg***
Heart rate [beats/min]	410 ± 24	441 ± 20	445 ± 39	420 ± 21
LVEDP [mmHg]	2.12 ± 0.20	3.99 ± 0.35**	2.52 ± 0.36	2.60 ± 0.36
LVSP [mmHg]	85.2 ± 2.3	99.8 ± 4.9*	84.2 ± 4.4	84.75 ± 4.18
LV +dP/dt [mmHg/s]	5368 ± 348	6009 ±514‡	5857 ± 618	4871 ±417
LV -dP/dt [mmHg/s]	-5465 ± 428	-5177 ± 605	-5355 ± 650	-4573 ± 384
WS_dias_posterior [mm Hg]	5.8 ± 0.5	7.7 ± 0.4*	6.7 ± 0.95	6.3 ± 1.41
WS_diastolic_septum [mm Hg]	5.1 ± 0.6	7.5 ± 0.5*	5.8 ± 0.6	7.16 ± 1.32
WS_systolic_posterior [mm Hg]	92.5 ± 9.8	96.8 ± 15.4	113 ± 7.7	108 ± 10.8
WS_systolic_septum [mm Hg]	77.8 ± 6.4	91.5 ± 7.5	106.5 ± 8.9	102 ± 10.6
**Echocardiographic parameters**	**LM_*Prog-Tg***	***Prog-Tg***	**LM_*LA-Tg***	***LA-Tg***
Ejection fraction [%]	62.8 ± 2	65.1 ± 2	60.6± 1.28	61.2± 4.01
EDD/BW [mm/g]	0.149 ± 0.033	0.155 ± 0.031	0.138 ± 0.013	0.142 ± 0.023
ESD/BW [mm/g]	0.094 ± 0.018	0.101 ± 0.026	0.089 ± 0.02	0.086 ± 0.03
EDSW/BW [mm/mg]	0.298 ± 0.020	0.397 ± 0.030*	0.247 ± 0.024	0.254 ± 0.048
EDPW/BW [mm/mg]	0.276 ± 0.025	0.363 ± 0.037*	0.258 ± 0.019	0.296 ± 0.036
ESSW/BW [mm/g]	0.051 ± 0.013	0.05 ± 0.008	0.038 ± 0.01	0.04 ± 0.01
ESPW/BW [mm/g]	0.036 ± 0.009	0.053 ± 0.009	0.037 ± 0.01	0.038 ± 0.01
E/e´ ratio	3.03 ± 0.10	2.92 ± 0.16	2.67 ± 0.08	2.77 ±0.07
IVRT [ms]	19.45 ± 0.5	17.7 ± 0.56*	18.33 ± 0.71	19.05 ± 0.7
MGTA [mmHg]	4.2 ± 0.21	4.7 ± 0.28	4.4 ± 0.26	4.5 ± 0.27

Supplemental Table 1. Hemodynamic and echocardiographic parameters. *Prog-Tg* and *LA-Tg* and corresponding littermates were analyzed (age >27 weeks; n=11 Wt, n=5 *LA-Tg*, n=6 *Prog-Tg*;). LVEDP, left ventricular end-diastolic pressure; LVSP, left ventricular systolic pressure; LV +dP/dt, maximal value of the first derivative of LV pressure; LV -dP/dt, minimal value of the first derivative of LV pressure; WS, wall stress; EDD, end-diastolic diameter; ESD, end-systolic diameter; EDSW, end-diastolic septal wall width; EDPW, end-diastolic posterior wall width; ESSW, end-systolic septal wall width, ESPW, end-systolic posterior wall width; E/e´, Ratio of E to e'; IVRT, isovolumic relaxation time; MGTA, mean gradient of transverse aorta. ‡ trend p<0.08,*p<0.05 and **p<0.01 Tg vs LM,. Unpaired Student´s *t*-test. Mean and error bars indicate SEM.

Supplemental Table 2. Primers used for quantitative real-time PCR analysis

Gene	Genbank accession number	Primer Sequences
Cdh5	NC_000074.6	Forward: 5'-CTGCTCACGGACAAGATCAGC-3'<br>Reverse: 5'-CTCTTTTGGCGATGGTGGGC-3'
Hprt	NM_013556.2	Forward: 5'-GCAGTCCCAGCGTCGTGATTA-3'<br>Reverse: 5'-TGATGGCCTCCCATCTCCTTCA-3'
Nos3	NM_008713.4	Forward: 5'-GCATGGGCAACTTGAAGAGTG-3'<br>Reverse: 5'-GCTGCCCACTTCCCAATTCT-3'
Col1a1	NM_007742.3	Forward: 5'-CTGACGCATGGCCAAGAAGA-3'<br>Reverse: 5'-ATACCTCGGGTTTCCACGTC-3'
Col3a1	NC_000067.6	Forward: 5'-TCCGGGAATAACGTCAGTC-3'<br>Reverse: 5'-GGAAGCCCATTTGCACCAGG-3'
Col4a5	NM_001163155.1	Forward: 5'-CCCAAGTGCACCAGCATAAC-3'<br>Reverse: 5'-AGAAGAACACCCATGGCAGG-3'
Actb	NM_007393.3	Forward: 5'-ACAGCTTCTTTGCAGCTCCT-3'<br>Reverse: 5'-TTGTCGACGACCAGCGCA-3'
Nppb	NM_008726.5	Forward: 5'-GGGCACAAGATAGACCGGAT-3'<br>Reverse: 5'-GCCAGGAGGTCTTCCTACAA-3'
Icam1	NC_000075.6	Forward: 5'-CAGATGCCGACCCAGGAGAG-3'<br>Reverse: 5'-CCGCTAGCTCCAAAACGCAG-3'
Icam2	NC_000077.6	Forward: 5'-GCTCACCGGCACAGAGGAGA-3'<br>Reverse: 5'-TATGGGCTTCAGGGGCACAG-3'
Acta2	NP_031418.1	Forward: 5'-GTACCACCATGTACCCAGGC-3'<br>Reverse: 5'-GAAGGTAGACAGCGAAGCCA-3'
Nos3 (promoter, ChIP)	NP_032739.3	Forward: 5'-CCCTCTAGCAGACAACCCAC-3'<br>Reverse: 5'-CTCTCAGATGCTGGCCTTCG-3'
Nos3 (gene body, ChIP)	NP_032739.3	Forward: 5'-AAGTGGGCAGCATCACCTAC-3'<br>Reverse: 5'-GGGACCAGGCCTAGAAACAC-3'

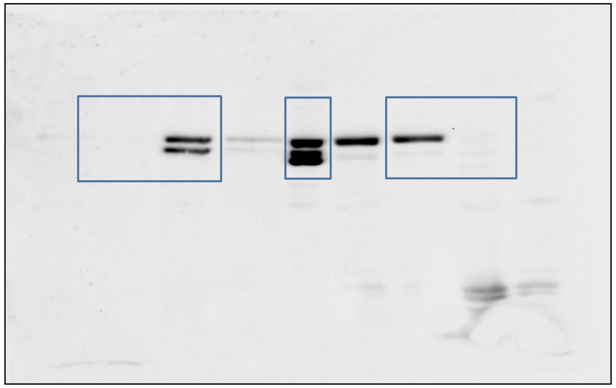

human lamin A

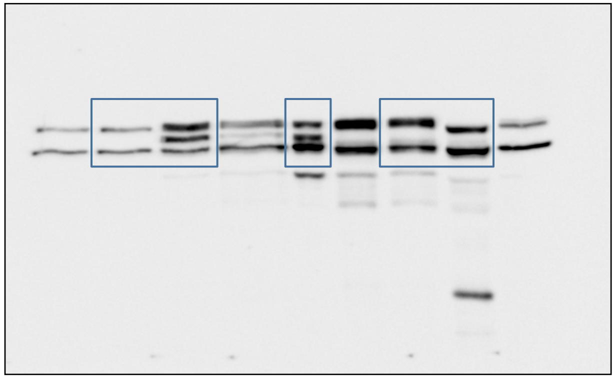

h+m lamin A

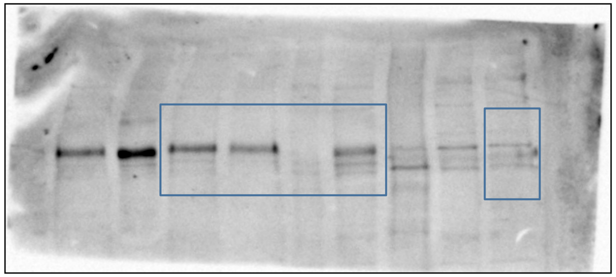

VE-cadherin

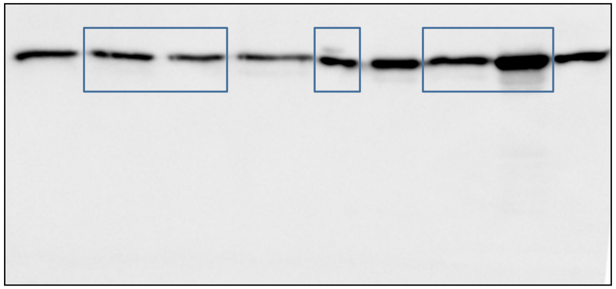

tubulin

* lower part of laminA (E1) blot separated and used for tubulin and also emerin (see Figure 5C)

Full unedited gels for Figure 1A.

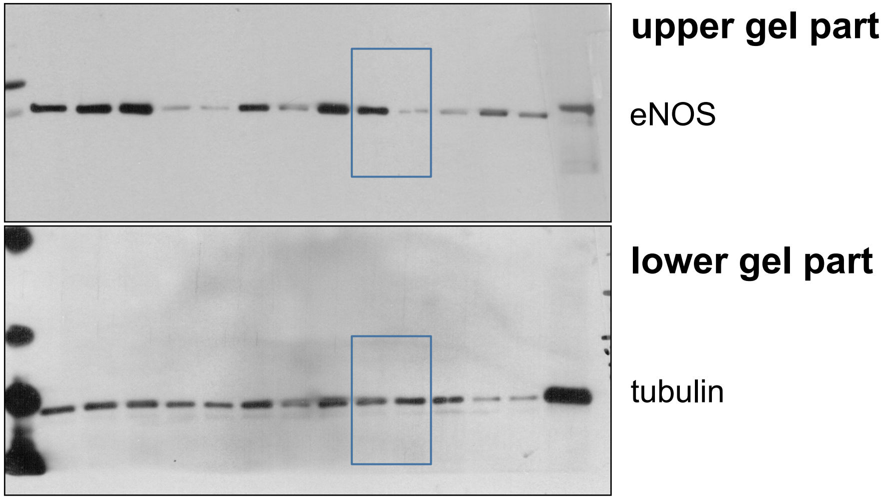

Full unedited gels for Figure 4B.

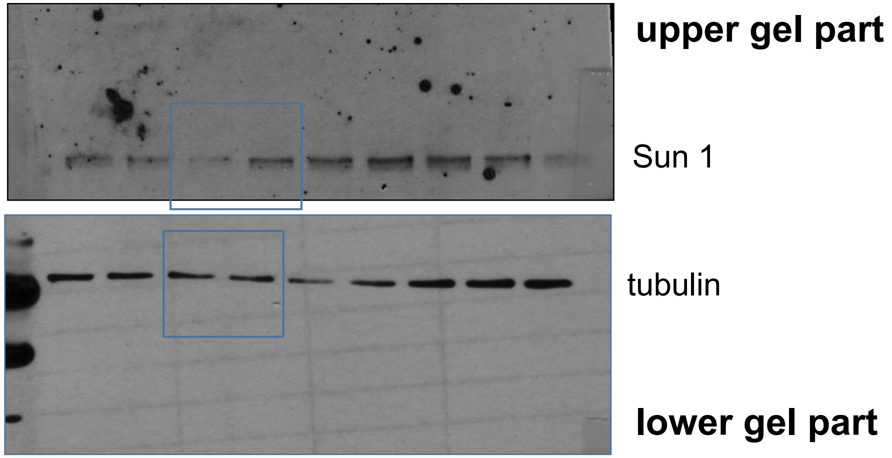

Full unedited gels for Figure 6B.

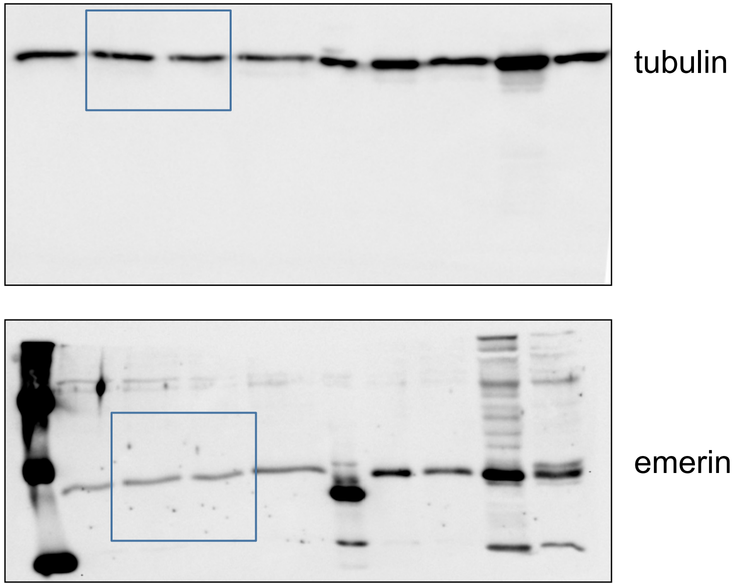

Full unedited gels for Figure 6C.

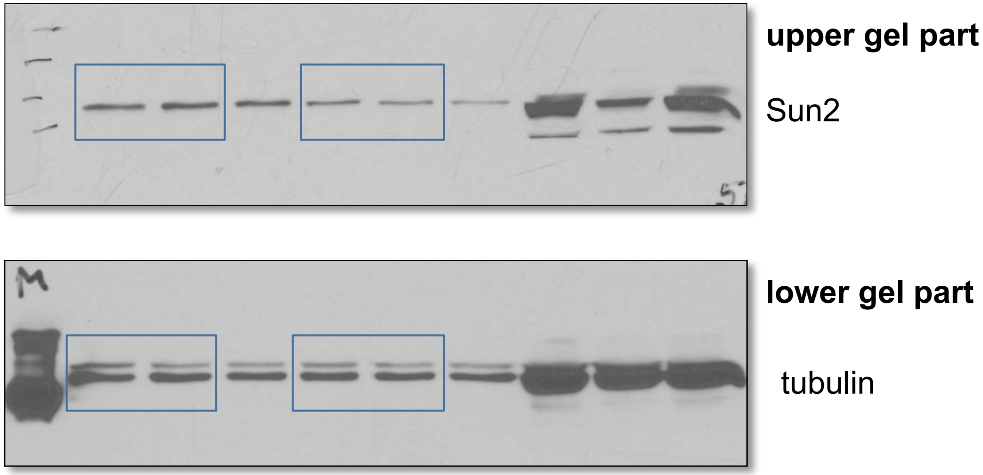

Full unedited gels for Supplemental Figure 4.
